# Mutational Study of the Tryptophan Tetrad Important for Electron Transfer in European Robin Cryptochrome 4a

**DOI:** 10.1021/acsomega.3c02963

**Published:** 2023-07-12

**Authors:** Anders Frederiksen, Corinna Langebrake, Maja Hanić, Georg Manthey, Henrik Mouritsen, Miriam Liedvogel, Ilia A. Solov’yov

**Affiliations:** † Institute of Physics, Carl von Ossietzky Universität Oldenburg, Carl-von-Ossietzky Strasse 9-11, Oldenburg 26129, Germany; ‡ Institute of Avian Research, An der Vogelwarte 21, Wilhelmshaven 26386, Germany; § Department of Biology and Environmental Sciences, Carl von Ossietzky University of Oldenburg, Carl-von-Ossietzky Strasse 9-11, Oldenburg 26129, Germany; ∥ Research Centre for Neurosensory Sciences, Carl von Ossietzky University of Oldenburg, Carl-von-Ossietzky Strasse 9-11, Oldenburg 26129, Germany; ⊥ MPRG Behavioural Genomics, Max Planck Institute for Evolutionary Biology, August-Thienemann-Str. 2, Plön 24306, Germany; # Department of Physics, Center for Nanoscale Dynamics (CENAD), Carl von Ossietzky University of Oldenburg, Ammerländer Heerstr. 114-118, Oldenburg 26129, Germany

## Abstract

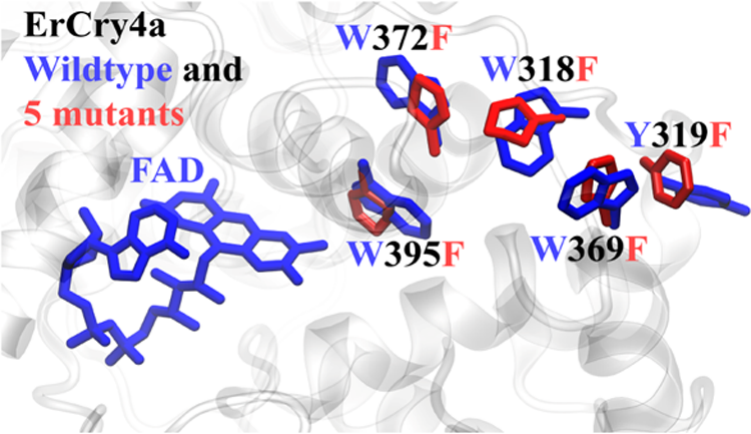

The ability of migratory birds to sense magnetic fields has been known for decades, although the understanding of the underlying mechanism is still elusive. Currently, the strongest magnetoreceptor candidate in birds is a protein called cryptochrome 4a. The cryptochrome 4a protein has changed through evolution, apparently endowing some birds with a more pronounced magnetic sensitivity than others. Using phylogenetic tools, we show that a specific tryptophan tetrad and a tyrosine residue predicted to be essential for cryptochrome activation are highly conserved in the avian clade. Through state-of-the-art molecular dynamics simulations and associated analyses, we also studied the role of these specific residues and the associated mutants on the overall dynamics of the protein. The analyses of the single residue mutations were used to judge how far a local change in the protein structure can impact specific dynamics of European robin cryptochrome 4a. We conclude that the replacements of each of the tryptophans one by one with a phenylalanine do not compromise the overall stability of the protein.

## Introduction

Each year, approximately one-fifth of the world’s known bird species embark on long-distance migration journeys utilizing the geomagnetic field of the Earth along with several other cues to find their way.^1−5^ Despite decades of research and a clear demonstration of the characteristics of the bird’s magnetic compass by cleverly designed behavioral experiments,^6−11^ it is remarkable that the mechanism of the magnetic sense in migratory birds is still not fully understood. Behavioral experiments have shown that the birds’ magnetic compass is an inclination compass that does not distinguish between the polarity of the magnetic field^6,12,13^ and requires ambient light to operate properly.^11,12,14−16^ Moreover, it has been demonstrated that the magnetic compass is linked to the birds’ visual system,^7,17−19^ suggesting that the primary magnetic field receptor could be located in the eyes of the animals, specifically in the retina, and that the compass is disrupted by radiofrequency magnetic fields,^20−24^ suggesting that the mechanism must be based on quantum mechanical principles related to electron spins.

Various mechanisms of how magnetoreception could work inside animals have been suggested. Currently, the best supported mechanism is based on a photosensitive quantum chemical reaction in a protein family called cryptochromes found in the bird’s eye.^2,15,25^ Cryptochromes, a multigene family of blue light photoreceptor proteins, are often discussed in the context of circadian rhythm regulation.^26−30^ However, a special member of the cryptochrome family, Cryptochrome 4a (Cry4a), has received much attention as a possible magnetic field receptor in migratory birds.^25,31−33^ The Cry4a protein shows high expression levels in the outer segments of the double cone photoreceptor cells in migratory birds’ eyes, and the Cry4a expression seems to be independent of the circadian clock.^31^ Upon activation with blue light, cryptochrome has the important ability to form so-called radical pairs, two unpaired electrons in a molecule, which could form the foundation for explaining its sensitivity to magnetic fields.^15,25,33−36^

The possibility that the formation of radical pairs in cryptochromes could be the mechanism responsible for magnetoreception in night-migratory songbirds has been investigated for decades^15,25,33−37^ although detailed studies of the hypothesis first gained traction after the turn of the millennium,^6,8,9,11^ especially in the 2010s^7,31,38^ when experimental results started backing up the theoretical predictions. The radical pair mechanism in cryptochrome involves light being absorbed by the flavin adenine dinucleotide (FAD) cofactor, which has been shown to be noncovalently bound in Cry4a structures.^39−41^ The absorbance of blue light by FAD in Cry4a triggers a fast electron transfer through the protein, resulting in the creation of a transient radical pair. Specifically, the electron transfer involves a tetrad of tryptophan amino acid residues.^15,25,33−35,37^ The radical pair is formed in a nonequilibrium process, meaning that even very weak interactions with external magnetic fields can have a substantial influence on the fate of the underlying spin chemistry.^15^ A recent landmark study by Xu et al.^33^ showed that electron transfers in night-migratory European robin (*Erithacus rubecula*) Cry4a (ErCry4a) in vitro involve a tetrad of tryptophan amino acid residues and that ErCry4a seemed to show a noticeably stronger response to a change in external magnetic fields compared to cryptochromes from nonmigratory birds like pigeon and chicken, even though the expected activation mechanism of all three avian Cry4a proteins is expected to be similar.^15,25,33^

**Figure 1:**
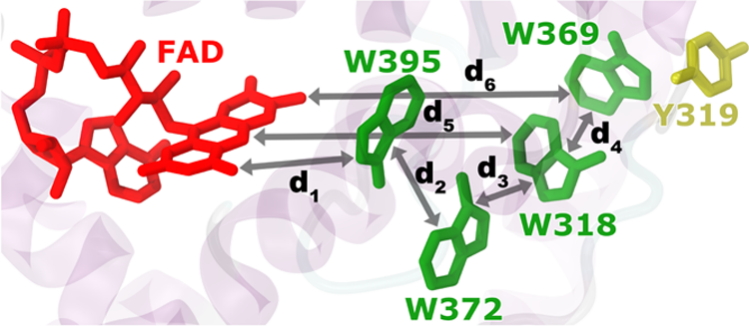
European robin cryptochrome 4a protein (transparent) featuring its tryptophan residues (green) and its tyrosine residue (yellow) with the FAD cofactor (red). The four tryptophan residues, involved in its activation, of Cry4a, namely, tryptophans 395, 372, 318, and 369, as well as tyrosine 319, were sequentially mutated in this study. The intramolecular distances d_1_–d_6_, important for functioning, are illustrated. Water has been omitted from the figure to aid clarity.

Furthermore, Xu et al.^33^ investigated the electron transfer through the tryptophan tetrad, highlighted in Figure 1, by introducing single amino acid mutations in the ErCry4a protein. Specifically, the four tryptophans important for the electron transfer were mutated individually into phenylalanine to selectively block the electron transfer at each step and thus to alter the activation process of the protein. Another potential candidate for electron transfer is tyrosine, which in earlier studies has been found to provide an alternative electron-transfer route in amphibian cryptochrome,^42^ and therefore Y319, located close to the last tryptophan in the tetrad, cannot be excluded as being a possible fifth link in the electron-transfer process.

An important question related to the ErCry4a mutants is whether these mutations significantly impact the overall structure of the protein, which is the focal question we investigate in the present paper. Specifically, our approach predicts how single amino acid mutations of the tryptophan tetrad as well as the Y319 residue in ErCry4a can influence the dynamics of the sites in the protein that are important for its activation as well as the dynamics of the whole ErCry4a protein. The investigations rely on molecular dynamics (MD) simulations as well as complementary phylogenetic analytical tools. More specifically, we have (i) simulated wild-type (WT) ErCry4a,^31,33^ the W395F, W372F, W318F, and W369F tryptophan mutants, and the Y319F mutant and carried out structural analyses based on the root-mean-square deviation and root-mean-square fluctuation of the important parts of ErCry4a and (ii) used a cross-species comparison on a phylogenetic level across 362 different avian species to compare our results with the natural variability in the tryptophan tetrad observed within WT bird Cry4a’s.

## Methods

### Phylogenetic Analysis

Cry4 sequences were isolated from the whole genomes provided by the B10K project,^43^ which includes 362 species that are quite evenly distributed across the whole avian phylogeny. An in-house script based on the blastn algorithm was used to isolate the sequence; the script was optimized to isolate the whole Cry4a sequence and excludes the possibility of accidentally isolating the closely related Cry1 or Cry2 sequences. Specifically, the target length for an exact sequence match (-word_size) was set to 10 as Cry4a has a variable sequence, and the chosen length permits for the best hits using mRNA data from NCBI and ErCry4a as a control query. The resulting hits were filtered according to the expected value (e-value; a measure for how likely a match by chance occurs; the lower the value, the more significant the match) and percent identity (i.e., similarity between the query and the target sequence, the higher the percent identity is, the more significant the match) as the initial quality measures. Genomic positions of the best hits were compared to the positions of the best hits of Cry1 and Cry2, and the correct order of exons was confirmed. All sequences were aligned with mafft,^44^ and the final codon alignment was generated using the MUSCLE algorithm in MEGA, which was then curated by hand by realigning misalignments, especially around exon borders, and incomplete codons were set to missing. For tests of selection, a consensus species tree with a 50% majority rule was generated based on 100 trees received from birdtree.org.^45^ As one species was not supported by the tree (*Apterix rowi*), it was excluded from further analysis.

The performed analyses particularly focused on the focal amino acid residues involved in intra-ErCry4a electron transfer by comparing the nuclear diversity across the avian clade. Test for selection pressure on the focal W318, W369, W372, and W395 residues as well as the Y319 residue was conducted through the site model of codeml.^46^ This program uses maximum likelihood to estimate the d*N*/d*S* (omega, ratio of nonsynonymous sites to synonymous sites) for each site, and hypotheses can be tested by comparing nested models, allowing for different complexities of the model. A value of omega <1 indicates negative (purifying) selection. Negative selection means that it is very likely that the given amino acid is the same between species (i.e., new mutations are very unlikely to establish themselves as they are deleterious). omega = 1 is equivalent to neutral selection, and omega >1 indicates positive selection. Positive selection means that it is more likely that the given amino acid varies between species (i.e., random mutations are more likely to establish themselves if they are beneficial for the organism). For this study, the nested codeml model M1 restricts omega to values ≤1, and codeml model M2 has no restrictions on omega and thereby allows for direct comparison of positively selected sites. To test for the significance of the M2 model, the negative log-likelihood values were compared with a likelihood-ratio test. The *p*-value was drawn from a χ^2^ distribution, and in the case of significance, the M2 model was accepted to explain the data.

### Molecular Dynamics Simulations

The structure of ErCry4a, illustrated in Figure 1, was adapted from an earlier study^33,47^ where it was constructed using pigeon (*Columba livia*) cryptochrome ClCry4a^48^ as a template for the structural homology modeling reconstruction.^49^ The single amino acid mutations were introduced into the stable ErCry4a structure using the built-in mutator tool in VMD,^50^ using the WT structure after its minimization and MD simulation.^33,48^

All structures were first neutralized with Na^+^ ions in water. Additional Na^+^ and Cl^–^ ions were then added to the system to achieve a NaCl concentration of 50 mM. The WT and mutated systems were simulated by using periodic boundary conditions with a box size of 92 Å × 105 Å × 100 Å in all simulations. The simulations were carried out using NAMD^51,52^ through the VIKING platform.^53^ The WT ErCry4a structure was minimized for 10,000 conjugated gradient steps and afterward equilibrated for 1 ns with the protein being restrained, followed by 2 ns with the backbone of the protein restrained, followed by another 2 ns with the system unrestrained. All of the equilibration simulations assumed a time step of 1 fs.

Mutated structures were created from the completed simulation of the WT ErCry4a; therefore, only 2 ns equilibration without any constraints was necessary for each studied mutation (see Table 1). The production simulation of the WT, as well as the mutated ErCry4a, was carried out for 500 ns. All hydrogen atoms in the simulated structures were placed at a fixed distance from the corresponding heavy atoms, allowing a time step of 2 fs to be used for all simulations. Simulations employed the CHARMM36 force fields for proteins with CMAP corrections^54−56^ along with an earlier parameterization of the FAD cofactor, which has been successfully employed in several earlier studies.^32,33,39,57−60^ All simulations assumed a temperature of 310 K, which was held constant through a Langevin thermostat and a 1 bar pressure, which was held constant through a Langevin barostat.^61−63^ Long-range van der Waals interactions were treated using a cutoff distance of 12 Å and a switching distance of 10 Å, and particle mesh Ewald^64^ was used to treat long-range electrostatic interactions with a grid size of 1 Å.

**Table 1:** Overview of the Performed WT and Mutational Simulations of ErCry4a, Indicated Jointly with the Production and Equilibration Simulation Duration

mutation type	simulation duration (ns)	equilibration simulation duration	reference
wild-type (WT)	500	10,000 minimization steps of 5 ns equilibration	(33)
W395F	500	2 ns	this study
W372F	500	2 ns	this study
W318F	500	2 ns	this study
W369F	500	2 ns	(33)
Y319F	500	2 ns	this study

## Results and Discussion

### Phylogenetic Analyses of Cry4a’s Tryptophan Tetrad

**Figure 2:**
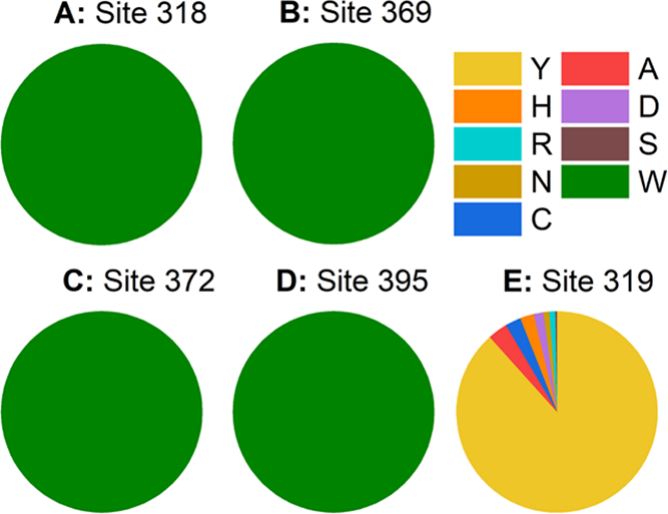
Distribution of amino acid states at the four investigated (A−D: the four tryptophan sites, E: the tyrosine site) sites across different bird species. The color code for the different amino acids is shown on the right in their one-letter shorthand notation. Distributions have been analyzed by using the methods described in the phylogenetic analysis in the Methods section.

The sequence of Cry4 was successfully isolated with at least 50% of the sequence present from 322 of the 362 species. The complete tryptophan tetrad (Figure 1) was present in all species for which Cry4 was successfully isolated. Though Cry4 sequences varied across the avian clade, the four tryptophans (W318, W369, W372, and W395 in ErCry4a) were 100% conserved in all species, both on the amino acid and on the codon level, which indicates extremely high conservation with neither synonymous nor nonsynonymous mutations occurring. Figure 2 shows the occurrence of the different residue types at each site for all of the considered bird species. This means that for ca. 35 million years, at least, no mutation has prevailed at any of these sites in any of the 322 different bird species from which the sequence information was available. This strongly suggests that the four tryptophans are extremely important for the function of Cry4 across bird species.

Site 319 (Figure 2E) was found to have the most variable residue composition, with eight different amino acids expressed across the 322 bird species; however, tyrosine is clearly the most abundant amino acid residue. The higher variability of the Y319 site is reflected by an omega value of 1.00, indicating that this site is evolving neutrally. Phylogenetic analyses indicate no conservation of tyrosines in migratory species; however, only one migratory passerine was identified without the tyrosine but instead a histidine at position 319.^65^

**Figure 3:**
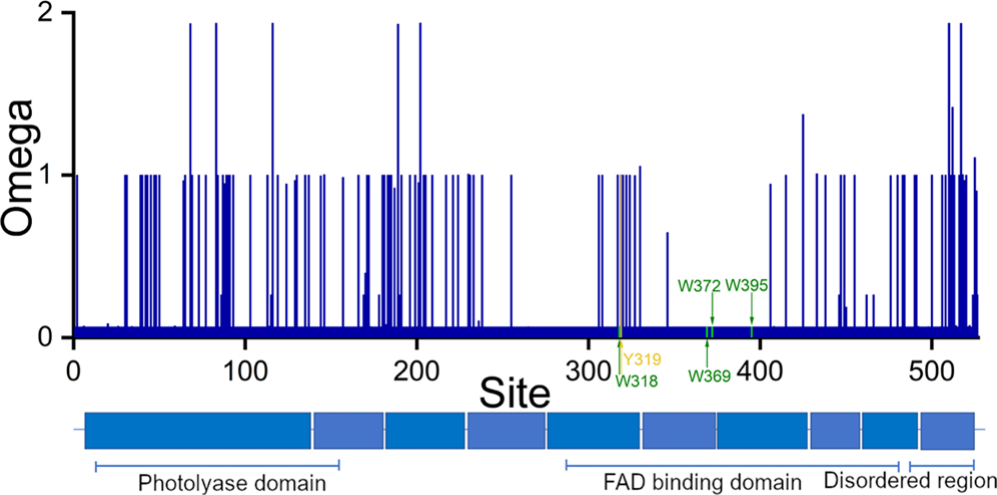
Evolutionary rate (omega) computed across the sequence of Cry4, with the five mutated sites highlighted in green (W, tryptophan) and yellow (Y, tyrosine). The FAD-binding domain is characterized by a high conservation of sites, resulting in strong purifying selection and low omega values. In contrast, the photolyase-domain and C-terminus (disordered region) show a higher variability through sites experiencing neutral selection and positive selection. Omega values = 1 are indicative of neutral selection, omega <1 indicates negative selection, and omega >1 highlights sites/areas under positive selection.

The test for positive selection (omega >1) through the empirical Bayes (NEB) was not significant, with a posterior probability of 0.0 for the 319 site. The sequence conservation at the tryptophan sites reflected in Figure 2A–D is also reflected by the strong purifying selection acting on the four sites (see Figure 3), where omega is 0.067 and the empirical Bayes (NEB) posterior probability is 0.0 when testing for an omega value above one for the four tryptophan sites. To sum up, the four focal tryptophan sites, with relevance for the radical pair formation process, have all been shown to be highly conserved across the avian clade, meaning that there is high selection pressure on having these tryptophans conserved.

### Structural Effects of Tryptophan to Phenylalanine Mutations in the Tryptophan Tetrad

Let us now consider how the mutations of the conserved tryptophan tetrad and the Y319 residue affect the structure and dynamics of ErCry4a locally and globally. Analyses of computational models of proteins should always start with ensuring that the stability of the investigated structures is within a certain threshold. Stability for protein structures can be estimated through the root-mean-square deviation (RMSD) value, which has been used successfully in earlier studies to determine the stability of protein models,^31,32,42,47,57,59,66−68^ and is defined as
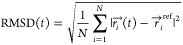
1Here, *N* is the number of backbone atoms in the protein, *r⃗*_*i*_(*t*) is the position vector of the *i*th backbone atom at a time instance, *t*, and *r⃗*_*i*_^ref^ is the position vector of the *i*th atom at some reference time point. Here, the structure at the beginning of the production simulation was considered as the reference. It should be noted that RMSD values for stable proteins of the size of ErCry4a are expected to be within a 2–4 Å range.^31,32,42,57,59,67,68^

**Figure 4:**
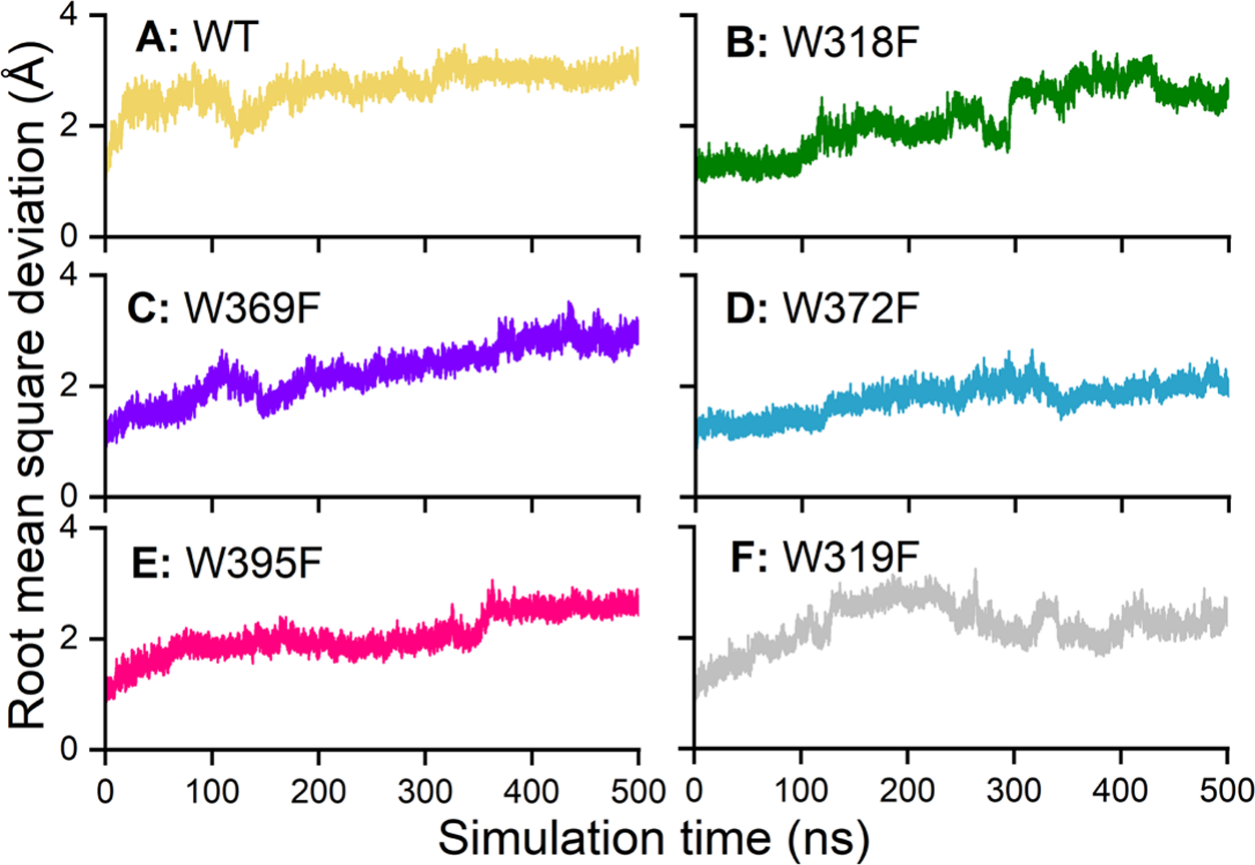
Root-mean-square deviation (RMSD) of the wild-type (A) and mutant (B−F) ErCry4a structures considered in the present study. The RMSD values have been calculated using eq 1, and the resulting values in the 2–4 Å range are the typical values for stable structures.

The time evolution of the RMSD values probed in this study is shown in Figure 4. The figure reveals that the studied structures appear to be sufficiently stable such that the introduced mutations do not give rise to any major changes in the stability of the mutated structures. The structures are assumed to be stable since the RMSD values for all studied systems do not exceed 4 Å, which is a comparatively low value for a protein comprising 487 amino acid residues.^31,32,42,57,59,67,68^ Moreover, in most cases, the RMSD time dependences flatten out, while a very shallow drift in, e.g., W369F mutant (Figure 4C) arises because of the intrinsically mobile phosphate-binding loop (residues 218–248);^60,69^ this part of the protein affects the overall RMSD values but does not change its overall stability. The high level of ErCry4a (WT and mutants) stability documents that the introduced substitutions of phenylalanine instead of tryptophan do not alter the protein structures to a larger extent than would be expected for a stable protein in vitro.

To assess whether the simulated ErCry4a and its mutants show dynamical differences, it is useful to compute the correlation times for the motion of the mutated residues. The correlation times for the center of mass (CoM) of the sites of interest have been calculated by means of the autocorrelation function defined as
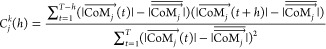
2Here, *k* indicates the simulated ErCry4a structure (WT, W318F, W369F, W372F, W395F, Y319F) and *j* denotes the site of interest (318, 369, 372, 395).  is the computed center of mass vector for site *j*, at a time instance *t*, while  denotes the length of the corresponding CoM vector.  is the average length of the CoM vector of site *j*, where the averaging has been performed over the entire MD trajectory.  is the CoM of site *j* at a lagged time instance *t* + *h*, and *T* is the total trajectory length. The autocorrelation function allows us to determine how long the specific CoM position of a residue remains correlated and compares a data series with itself for temporal intervals ranging between the lag time values of *h* = 0 and *h* = *T* – 1; for *h* = 0, the autocorrelation function is equal to unity per definition. In more general terms, the autocorrelation function permits us to quantify the time interval at which the specific motions inside the protein can be concluded as being statistically independent. This time interval is also known as the correlation time (τ_c_) and can be found by fitting the correlation function with a sum of exponential decay functions as
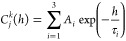
3Here, a sum of 3 exponential functions has been employed, where *A_i_* denotes the weight of the *i*th exponential decay, satisfying the normalization condition ∑_*i*=1_^3^*A*_*i*_ = 1. τ_*i*_ in eq 3 are the correlation times for each exponential function (see Figures S7–S12). The cumulative correlation times τ_c_ can then be estimated from the τ_*i*_ values as
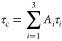
4The correlation times τ_c_ of the four residues (318, 369, 372, 395) were established for ErCry4a and its mutants and are compiled in Table 2. It can be observed that the correlation times for the CoM motion of the investigated residues are significantly smaller than the overall lengths of any performed MD simulation carried out in this study (see Table 1).

**Table 2:**
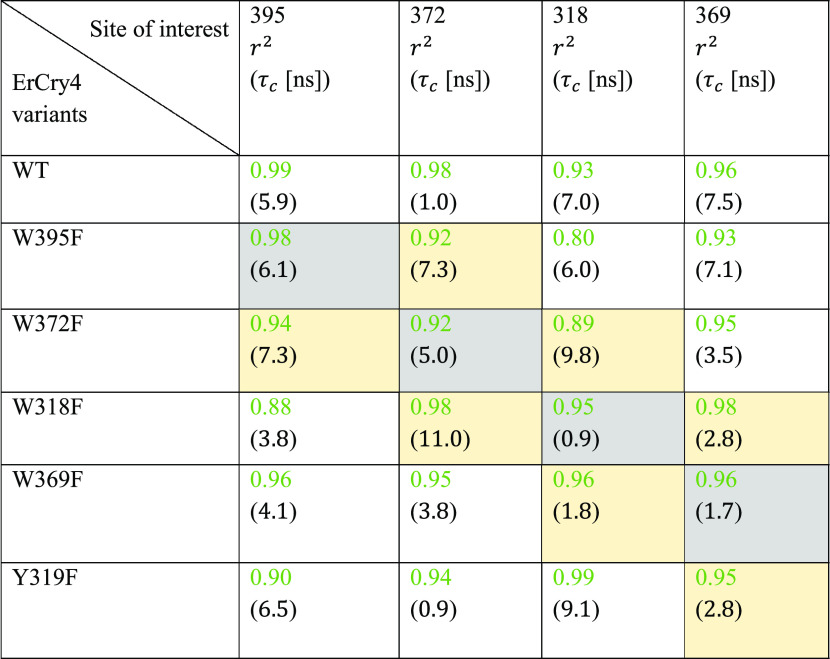
Green Numbers Are Residual Sum of Squares (*r*^2^) Values Corresponding to the Fitted Distributions of RMSF Values (See Figures S13–S16 for the Fitted Data) for the 10 Residues Surrounding the Investigated Tryptophan Sites for the WT ErCry4a and Its Mutants^a^

a The correlation times (τ_c_), see eq 4, for the center of mass motion of the studied residues are shown in parentheses. The correlation times were calculated for the center of mass motion of the backbone atoms of the site of interest and all amino acid residues within a 10 Å radius from the corresponding site. The entries with the result values for the corresponding mutated sites have been highlighted in gray, while the data for the neighboring sites have been marked in yellow.

The shortest correlation times are observed for the 372 site of the WT and the Y319F mutation, as well as in the case of the 318 site of the W318F mutation and the 372 site in the Y319F mutation, whereas the longest (about 10 ns) is observed for the 372 site of the W318F mutation. Interestingly, the W395F mutation is the only mutation that shows a consistent correlation time for the four mutant simulations, with the correlation times being approximately equal to 6–7 ns, indicating that the mutation does not impose any major changes to the internal motion of the tryptophan tetrad. The correlation times for the other sites show a different behavior; here, one can observe a noticeable change in the correlation times of the CoM motion of the studied residues when the residue is substituted with phenylalanine. The correlation time of the 372 residue in the W372F mutant increases nearly 5-fold compared to the WT ErCry4a, while the correlation times for the mutated sites of W318F and W369F decrease by a factor of 7 and 5, respectively, in the respective mutated configuration. The correlation times obtained from the fits to the simulated data are expected to represent the underlying dynamics of the studied residues within at least an order of magnitude. This conclusion is supported by the high *r*^2^ values, reflecting the quality of the fits (see Table 2).

The results in Table 2 should therefore give a characteristic time frame for the correlated motions of the studied residues and also indicate cases where dynamics is possibly affected by point mutations. Table 2 also shows that the tryptophan sites that neighbor the mutated site appear to be affected in the case of residues 372, 318, and 369 (see entries marked in yellow in Table 2). The results in Table 2 therefore suggest that a substitution of a tryptophan residue to phenylalanine leads to a disturbance of the hydrogen-bonding network around the mutated residue that affects their local dynamics. Although noticeable changes in the CoM correlation times could be recorded at the local level, these differences are still not likely to be critical for the dynamics of the whole mutated protein since they seem to have a rather local nature.

The correlation times in Table 2 indicate that the WT and mutated ErCry4a trajectories each could be subsampled into 20 statistically independent blocks of 25 ns, which allows the statistical analysis to significantly increase the statistics of the analysis.

**Figure 5:**
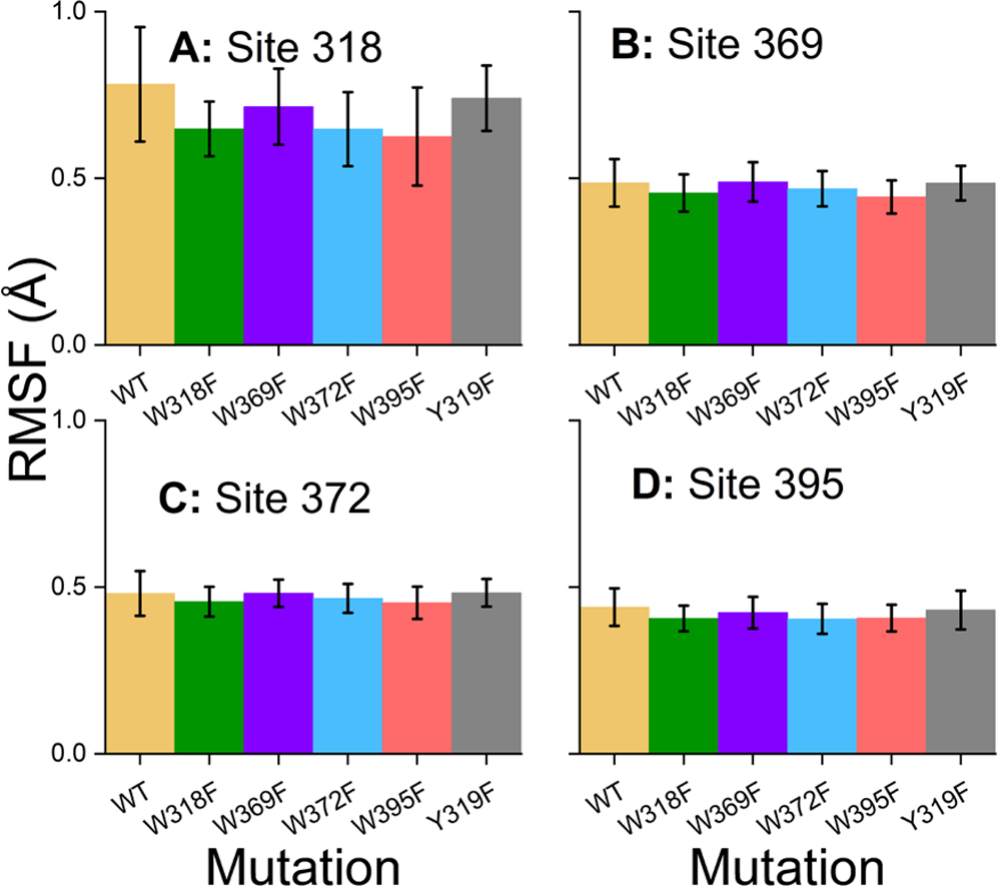
Root-mean-square fluctuations (RMSFs) of ErCry4a (eq 5) at the four tryptophan sites (A: site 318, B: site 369, C: site 372, and D**:** site 395) for the four tryptophan mutations considered in this study. The bar in each plot represents the average fluctuation of the site of interest in the given structure (WT or mutant), while the error bars signify the standard deviation of the fluctuations caused by the 10 residues around the four studied sites. Overall, no significant differences among the individual mutations could be noted.

Root-mean-square fluctuation (RMSF) analysis provides an important measure of how much the backbone atoms of each individual amino acid fluctuate over the course of the trajectory and is defined as
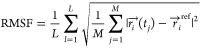
5Here, *M* is the total number of frames in the MD trajectory fragment (5000 for all simulations), *L* = 20 is the number of trajectory blocks used in the subsampling scheme, *r⃗*_*i*_(*t*_*j*_) is the position vector of the *i*’th backbone atom at a time instance, *t*_*j*_, and *r⃗*_*i*_^ref^ is the position of the atom used as a reference for the RMSF calculation. In this study, the initial configuration of the system prior to the production simulation was used as a reference. Figure 5 shows the computed RMSF values at the four tryptophan sites (W318, W369, W372, and W395) for the six studied ErCry4a variants (WT, W318F, W369F, W372F, W395F, Y319F). The error bars in Figure 5 indicate the standard deviations of the RMSF values with an account of the 10 residues around a site of interest; see Figures S13–S16 in the Supporting Materials (SMs).

Figures S13–S16 show the distributions of the RMSF values and the corresponding Gaussian fits that were used to compute the standard deviations; the double Gaussian fits for the WT, W395F, and Y319F in Figure S13 due to the presence of slight residual higher-amplitude fluctuations of some amino acids around the 318 site are noted. The associated residual sum of squares, *r*^2^, values of the Gaussian fits are compiled in Table 2 and demonstrate that the Gaussian distributions yield a very good approximation resulting in all *r*^2^ values greater than 0.8 (Table 2) in all of the fits. It should be noted that an *r*^2^ value of 0.8 indicates that the corresponding Gaussian distributions deliver fits with an 80% accuracy. Figure 5 demonstrates that no major statistical differences between the studied mutations could be observed in the RMSF profiles if one takes the statistical uncertainties into account.

**Figure 6:**
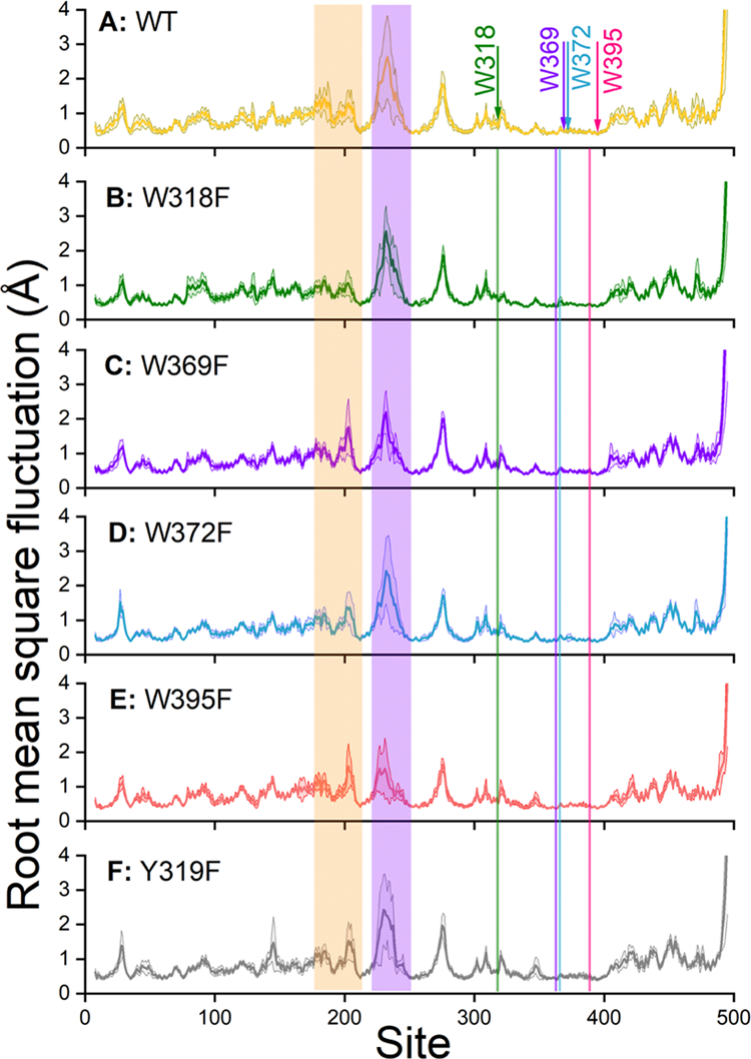
Averaged root-mean-square fluctuations (RMSFs) computed for each individual ErCry4a mutation introduced in this study (eq 5) with the standard deviation of the averaging given by the semitransparent area around each data set. The mutated sites of the tryptophan tetrad have been marked with vertical arrows, and two regions (helices 8–10, residues 174–210 (light brown) and phosphate-binding loop, residues 218–248 (purple)) with varying RMSFs between the different simulations have been indicated.

Figure 6 shows the overall fluctuations in ErCry4a WT and in the studied mutants and reveals that the five mutation sites (318, 319, 369, 372, and 395) do not lead to any major changes in the fluctuations inside the protein variants. It can be seen that the differences between the WT structure and the mutated structures are essentially constrained within the statistical deviation. Figure 6 also reveals that certain regions in ErCry4a and its mutants are rather rigid, while some regions (highlighted) are more flexible. The region of ErCry4a containing the tryptophan tetrad, residues 318–395, turns out to be one of the most stable regions for the entire protein. This stability might be essential for the underlying electron transfer responsible for the ErCry4a activation^25,33^ as the electron transfer cannot be completed efficiently if the distance between the donor and acceptor sites varies significantly.^42,70^

**Figure 7:**
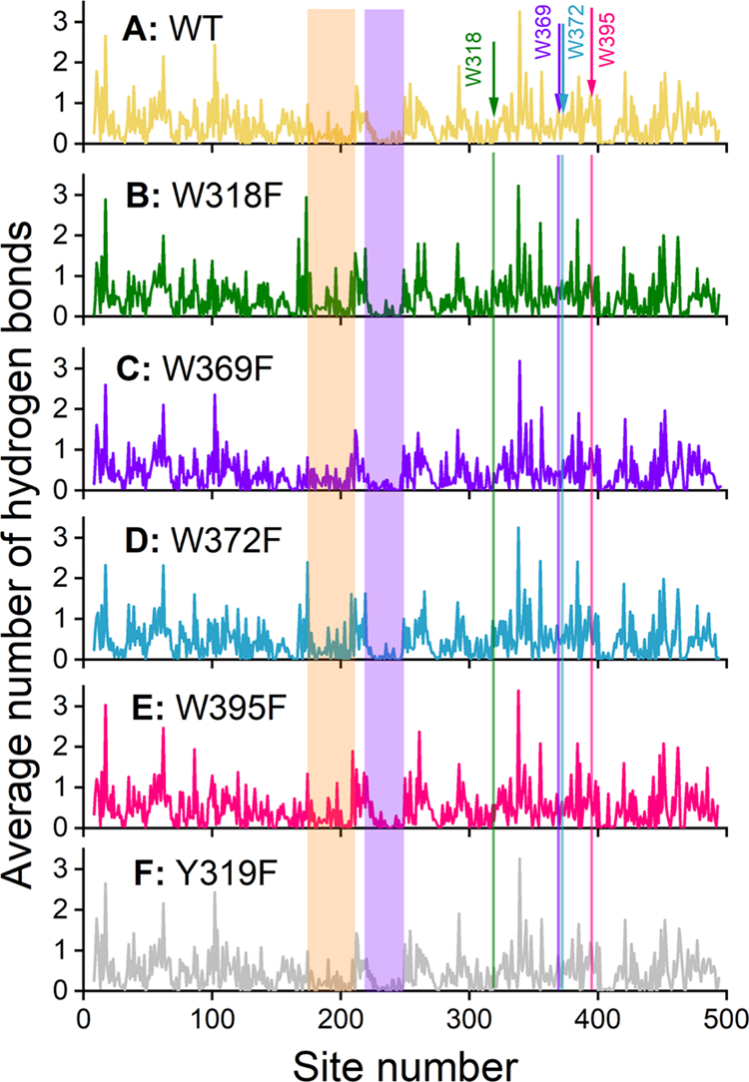
Average number of hydrogen bonds in WT ErCry4a and the five mutations considered. The mutated sites of the tryptophan tetrad have been marked with vertical arrows, and the two regions (residues 174–210 (light brown) and 218–248 (purple)) also indicated in Figure 6 have been highlighted.

In order to further investigate the differences in fluctuations of the different sites in Figure 6, it is reasonable to analyze the hydrogen bonds for each residue. The analysis was accomplished using a parallelized hydrogen bond script^71^ that recorded all possible hydrogen donor and acceptor pairs within 3 Å of each other, assuming a maximal deviation angle of 20°. The total number of possible hydrogen bonds was calculated for each residue and was averaged over the simulation time. The resulting number of average hydrogen bonds per residue is shown in Figure 7, which shows that the two areas with the largest internal fluctuations (marked areas in Figure 6) are the areas that have the least hydrogen bonds. On the contrary, the part of the protein around the tryptophan tetrad is the region with the highest number of average hydrogen bonds. This observation indicates that the number of hydrogen bonds in a protein is highly correlated to its rigidity. Table 3 summarizes the acceptor and donor residues forming hydrogen bonds at the ErCry4a sites of interest. In the listed cases, the hydrogen bonds were formed with the four tryptophans involved in the ErCry4a activation, and their corresponding partners have been identified. The hydrogen bonds have been estimated through a Python script.^71^ Quite surprisingly, it is observed that certain hydrogen bonds are preserved among the different mutations, which contrasts earlier studies that reported notable deviations in the presence of hydrogen bonds among different mutations^72,73^

**Table 3:** Summary of the Acceptor Residues That Form Hydrogen Bonds with the Principal Tryptophans of the Electron-Transfer Chain in ErCry4 (Donor Residues)^a^

WT			W318F			W369F			W372F			W395F			Y319F		
donor	acceptor	%	donor	acceptor	%	donor	acceptor	%	donor	acceptor	%	donor	acceptor	%	donor	acceptor	%
W318 (O)	K320 (NZ)	4	F318 (O)	K320 (NZ)	1	W318 (O)	K320 (NZ)	8	W318 (O)	K320 (NZ)	1	W318 (O)	K320 (NZ)	3	W318 (O)	K320 (NZ)	3
W369 (NE1)	C317 (O)	32	W369 (NE1)	C317 (O)	18	F369 (NE1)	C317 (O)	37	W369 (NE1)	C317 (O)	30	W369 (NE1)	C317 (O)	38	W369 (NE1)	C317 (O)	36
W369 (O)	Y319 (N)	51	W369 (O)	Y319 (N)	53	F369 (O)	Y319 (N)	0	W369 (O)	Y319 (N)	54	W369 (O)	Y319 (N)	48	W369 (O)	F319 (N)	49
W372 (O)	M376 (N)	41	W372 (O)	M376 (N)	43	W372 (O)	M376 (N)	42	F372 (O)	M376 (N)	39	W372 (O)	M376 (N)	35	W372 (O)	M376 (N)	36
W395 (N)	N391 (O)	23	W395 (N)	N391 (O)	23	W395 (N)	N391 (O)	24	W395 (N)	N391 (O)	23	F395 (N)	N391 (O)	25	W395 (N)	N391 (O)	33
W395 (O)	S399 (N)	8	W395 (O)	S399 (N)	8	W395 (O)	S399 (N)	7	W395 (O)	S399 (N)	11	F395 (O)	S399 (N)	12	W395 (O)	S399 (N)	8
W395 (O)	S399 (OG)	57	W395 (O)	S399 (OG)	54	W395 (O)	S399 (OG)	63	W395 (O)	S399 (OG)	56	F395 (O)	S399 (OG)	60	W395 (O)	S399 (OG)	63

a Residue numbers and the names (CHARMM nomenclature) of the atoms participating in hydrogen bonds are indicated. The percentage shows the fraction time of the 500 ns simulations, where the corresponding hydrogen bonds could be observed.

**Figure 8:**
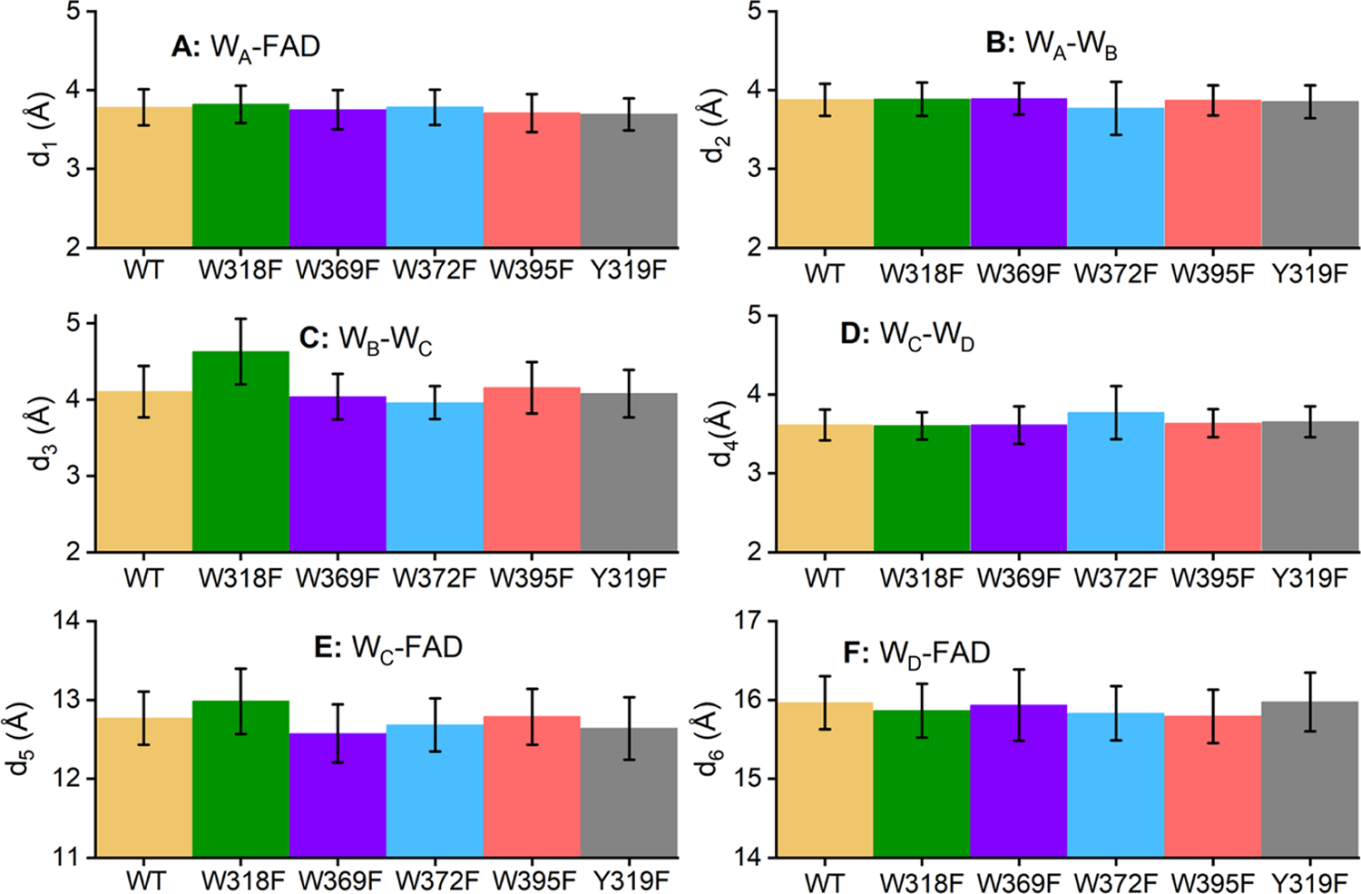
Intramolecular distances (A−F) are introduced in Figure 1 for the different ErCry4a WT and mutant structures. Here, the bar signifies the distance, averaged over the span of the 500 ns long simulations, measured between the different parts of ErCry4a. The error bar represents the standard deviation of the corresponding distance obtained over the course of the 20 subsampled parts of the MD simulations. Figures S1–S5 in the SM show the probability density distributions of the computed distances that were used to fit the data.

The influence of the mutations on the intermolecular distances relevant for the cascade of electron transfers (d_1–4_) (see Figure 1) has been summarized in Figure 8. Figure 8 shows that the introduced mutations do not significantly affect the intermolecular distances between the tryptophans. One, however, notices a slight peculiarity for the W_B_–W_C_ distance (see Figure 8C) in the case of the W318F and W372F mutations.

The results in Figure 8 have been calculated fitting the data to a Gaussian distribution; the corresponding statistical fits are provided in Figures S1–S5. It can be observed in Figure 8 that the distances important for electron back transfer (d_5_ and d_6_) are also not significantly influenced by the studied mutations.

Lastly, it is seen that the standard deviations of the distances in Figure 8 are rather small, which is not a surprising result given that the region around each conserved tryptophan is very stable (see Figure 6) and the deviation of distances has been found to be very small in similar studies.^33,70^

**Figure 9:**
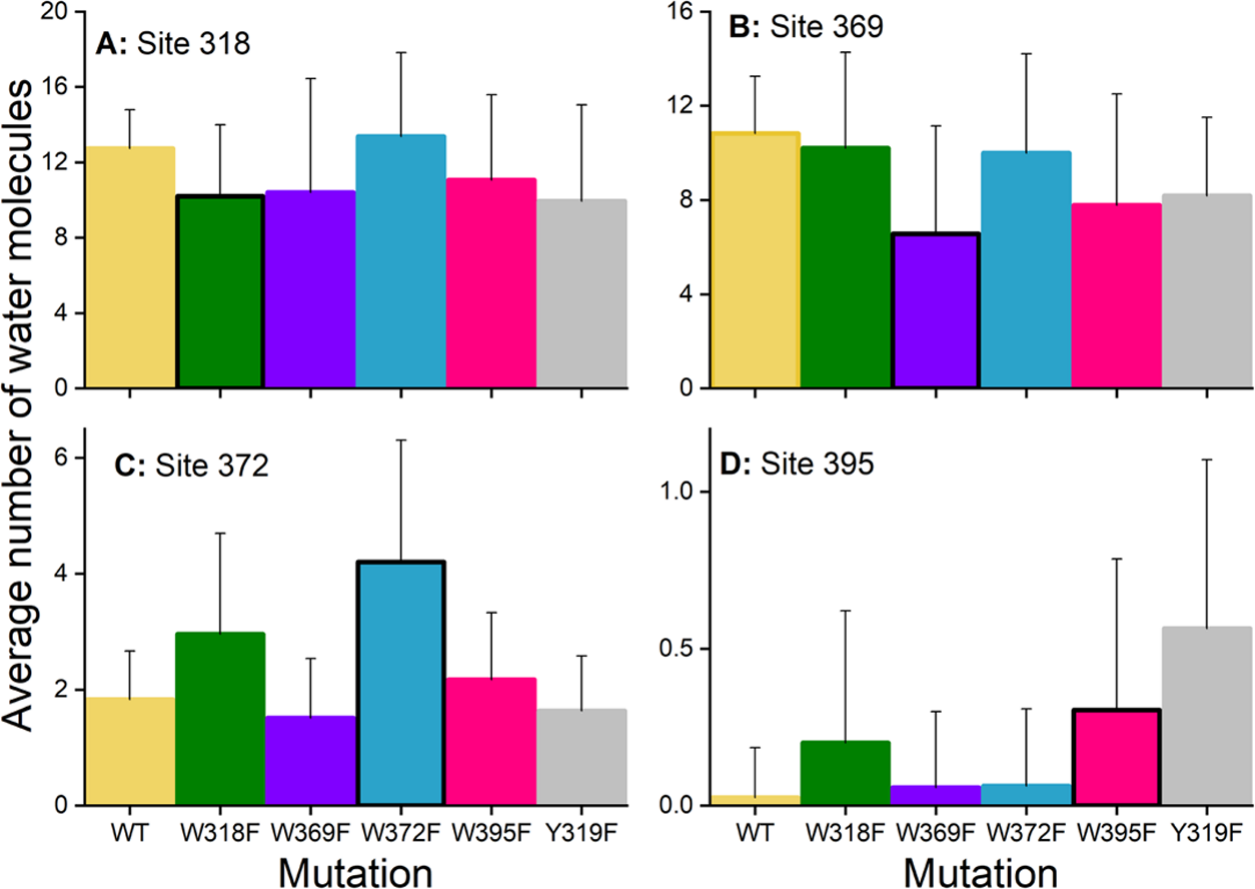
Number of water molecules found at each tryptophan site of interest (A−D) for the WT and five mutant ErCry4a structures, averaged over the span of 500 ns simulations. Water molecules within a 5 Å radius from the corresponding site were counted. The black outline highlights cases where the respective sites of each subplot experienced a W → F mutation.

An interesting question that remains open from the analysis of differences in distances between the tryptophan residues concerns the water exposure of the four studied sites in ErCry4a, which is related to the hydrophilic–hydrophobic interactions at each site. These interactions were reported earlier to influence charge mobility in proteins.^74^ To characterize the difference in water exposure of the tryptophan sites imposed by the mutations, the water molecules found around each of the four sites have been counted. Figure 9 shows the resulting amount of water molecules that appear along the tryptophan tetrad. Not surprisingly, there is a much lower amount of water molecules around the sites nearest to the FAD, namely, sites 372 and 395 (Figure 9C,D). Figure 9A shows that the amount of water near site 318 does not change significantly among the different ErCry4a mutations, while the other three sites feature more prominent changes, especially once the corresponding tryptophan is modified. A surprising effect is the change in water molecules at site 395 (Figure 9D) when mutation Y319F is introduced. This result is especially interesting since no direct interaction between W395 and Y319 could be resolved; see Table 3. The differences in water exposure stemming from introducing a mutation, as seen in Figure 9B–D, are surprising as both phenylalanine and tryptophan have earlier been categorized as hydrophobic amino acids^75^ and one would thus expect them to have a similar affinity for water exposure, leading to a small expected difference, such as that seen in Figure 9A.

**Figure 10:**
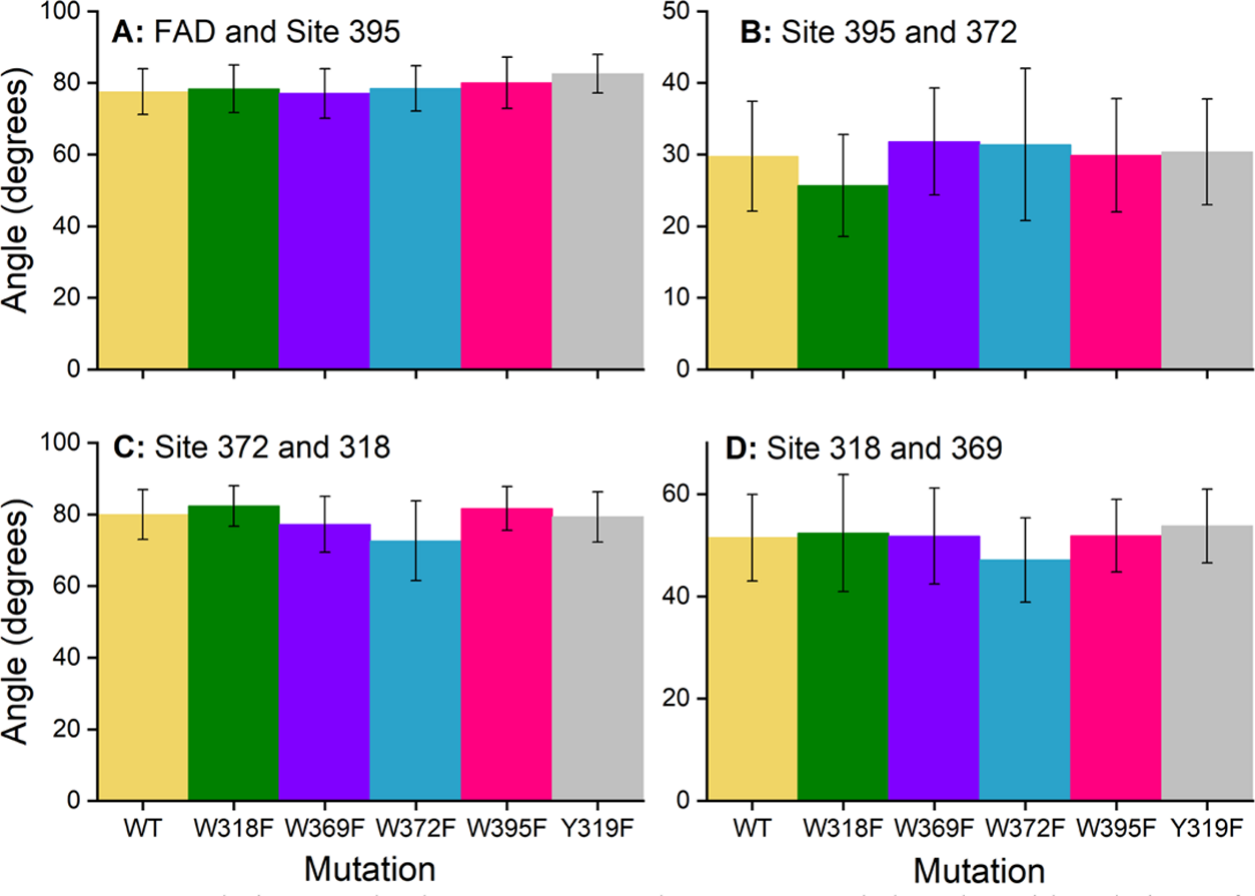
Relative angle between two planes spanned by the side chains of two neighboring sites in the electron chain among the difference mutations or wild-type ErCry4a protein studied. The bars in the figure represent the average angle, calculated by averaging over the entire span of the 500 ns trajectory for each of the simulations, with the error bars representing the standard deviation.

To investigate the possible stacking interaction between the four investigated tryptophan sites, the relative angles between pairs of aromatic residues at neighboring sites have been investigated. These angles have been estimated by using three atoms from the aromatic side chains of each respective tryptophan, phenylalanine, or FAD cofactor to define the plane associated with each residue, and an angle between the planes attributed to the neighboring ones was computed for each frame of the MD simulations. The resulting time-averaged relative angles are summarized in Figure 10. Figure 10 shows that the mutations do not introduce any major differences in the relative orientation of any neighboring sites, which suggests that the blocking of electron transfers found by Timmer et al.^76^ should not be caused by any reorientation of the mutated residues.

It can therefore be concluded that the effects on different parameters important for the electron-transfer processes, linked to the radical pair mechanism of the Cry4 proteins,^33,42,59^ are not expected to be majorly influenced by the structural changes introduced from the mutations. The mutational procedure could therefore be a robust approach to studying the kinetics of the individual electron-transfer steps in ErCry4a. It should be noted that the presented data do not take into account the free-energy differences or reorganization energy differences introduced by the mutation, both highly important for the electron-transfer process but not for the structure. Furthermore, the differences of the side chains between a tryptophan and a phenylalanine will influence parameters such as the free energy and reorganization energy, which might explain the observed difference in magnetic response of the WT ErCry4a cryptochrome versus the mutated cryptochrome found by Xu et al.,^33^ nor the blocking of electron transfers observed by Timmer et al.^76^

## Conclusions

Our study has utilized a combination of phylogenetic research with extensive molecular dynamics analyses, which builds a crucial foundation for further studies in the evolution of avian cryptochromes. The study further emphasizes that the four investigated tryptophans are all highly conserved in the avian clade, while tyrosine 319, which is speculated to be important for further signaling,^25^ shows a higher variability within the avian clade. Furthermore, the performed molecular dynamics analyses show that the introduced mutations of the highly conserved tryptophan tetrad and tyrosine sites in ErCry4a produce only minor differences in the investigated protein structures and dynamical traits. The high level of ErCry4a (WT and mutants) stability documents that the introduced mutations do not alter the protein structures to a larger extent than would be expected in a stable protein in vitro and hence suggests that the investigated sites could have a well-defined structural function. The presented combination of phylogenetic analyses and molecular dynamics simulations is instrumental and could become an important tool for investigating similarities and differences in avian cryptochromes that would aid our understanding of how certain bird species obtained the ability to sense magnetic fields during evolution. The study thus reveals that mutations around the particular tryptophan residues inside the Cry4 family of proteins, which are thought to be essential for the protein’s functioning, keep the protein dynamics intact. This includes the interprotein hydrogen-bonding network and the relative orientation between the tryptophans. Though most characteristics are kept intact, there seems to be an increase in exposure to water in some cases, although the overall results suggest that the studied mutated cryptochromes should largely preserve their structure and functionality, except for the intentionally blocking electron transfer along the investigated tryptophan tetrad. The study therefore concludes that the W → F mutation is a powerful tool for blocking one specific process in ErCry4a and that such mutants will help us gain a more fundamental understanding of the activation process of the protein.

## Supporting Information Available

Fitting plots of the distance distributions used for generating average and standard deviations for Figure 8, fitting of correlation functions used to estimate correlation times found in Table 2, and fits of RMSF distributions used to generate averages and standard deviations used for Figure 5 (PDF)
