# Supplementary material for: Mutational Study of the Tryptophan Tetrad Important for Electron Transfer in European Robin Cryptochrome 4a
Source: ACS Omega. 2023 Jul 12;8(29):26425–36. doi: 10.1021/acsomega.3c02963 (PMC10373462; doi:10.1021/acsomega.3c02963)
Supplement: Supplementary file 1 — ao3c02963_si_001.pdf [file ao3c02963_si_001.pdf]

Mutational study of the tryptophan tetrad important for electron transfer in European robin cryptochrome 4a (Supporting materials)

Anders Frederiksen[1], Corinna Langebrake[2], Maja Hanić[1], Georg Manthey[1,2], Henrik Mouritsen[3,4], Miriam Liedvogel[2,3,5] and Ilia A. Solov'yov[1,4,6*]

[1]Institute of Physics, Carl von Ossietzky University of Oldenburg, Carl-von-Ossietzky Strasse 9-11 Oldenburg 26129, Germany

[2] Institute of Avian Research, An der Vogelwarte 21 Wilhelmshaven 26386, Germany

[3] Department of Biology and Environmental Sciences, Carl von Ossietzky University of Oldenburg, Carl-von-Ossietzky Strasse 9-11 Oldenburg 26129, Germany

[4] Research Centre for Neurosensory Sciences, Carl von Ossietzky University of Oldenburg, Carl-von-Ossietzky Strasse 9-11 Oldenburg 26129, Germany

[5] MPRG Behavioural Genomics, Max Planck Institute for Evolutionary Biology, August-Thienemann-Str. 2, Plön 24306, Germany

[6] Department of Physics, Center for Nanoscale Dynamics (CENAD), Carl von Ossietzky University of Oldenburg, Ammerländer Heerstr. 114-118, 26129 Oldenburg

[*] *Email: ilia.solovyov@uni-oldenburg.de*

Supporting figures

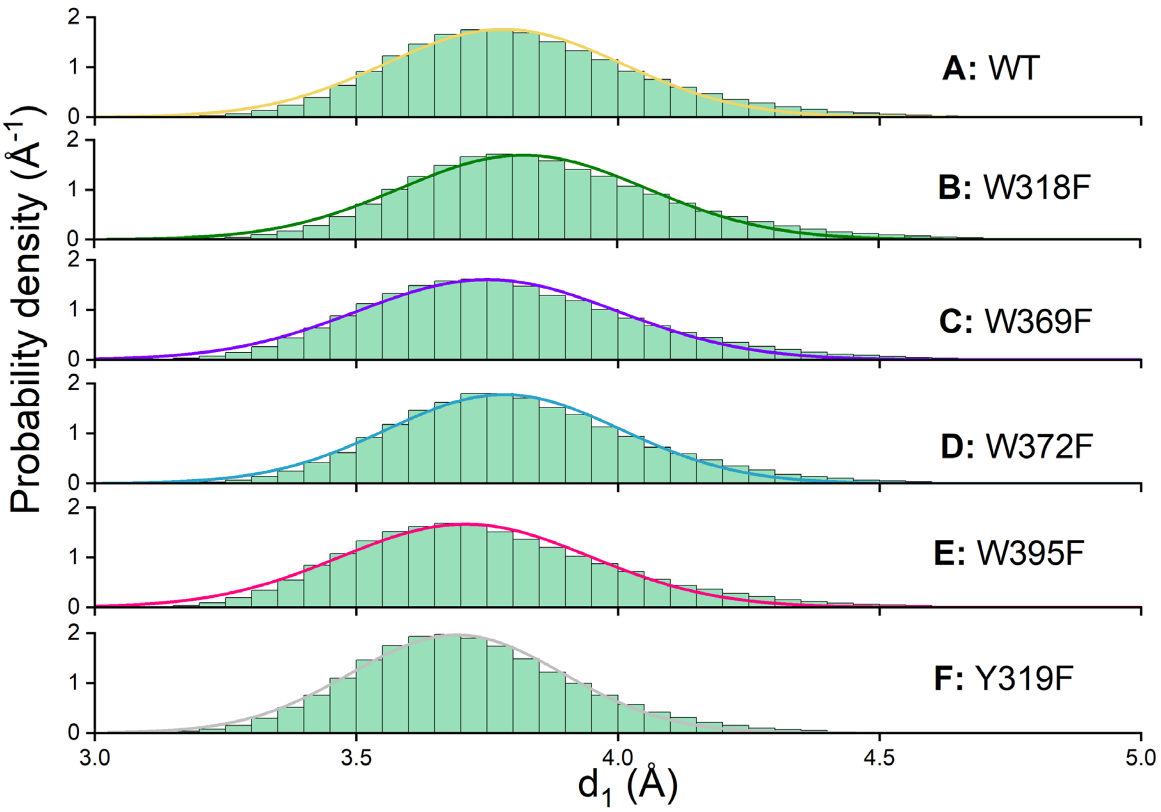

Figure S1: Probability distributions of the intramolecular contact (edge-to-edge) distance between FAD and W_A in ErCry4a, see Fig. 1. The distributions are shown for the wildtype ErCry4a (**A**) and mutations considered in this study (**B-F**). The distances were sampled every 5 ps of the production simulation yielding a total of 100,000 data points. The fitted distributions (lines) correspond to the single Gaussian fit functions, fitted utilising the Levenberg-Marquardt algorithm.

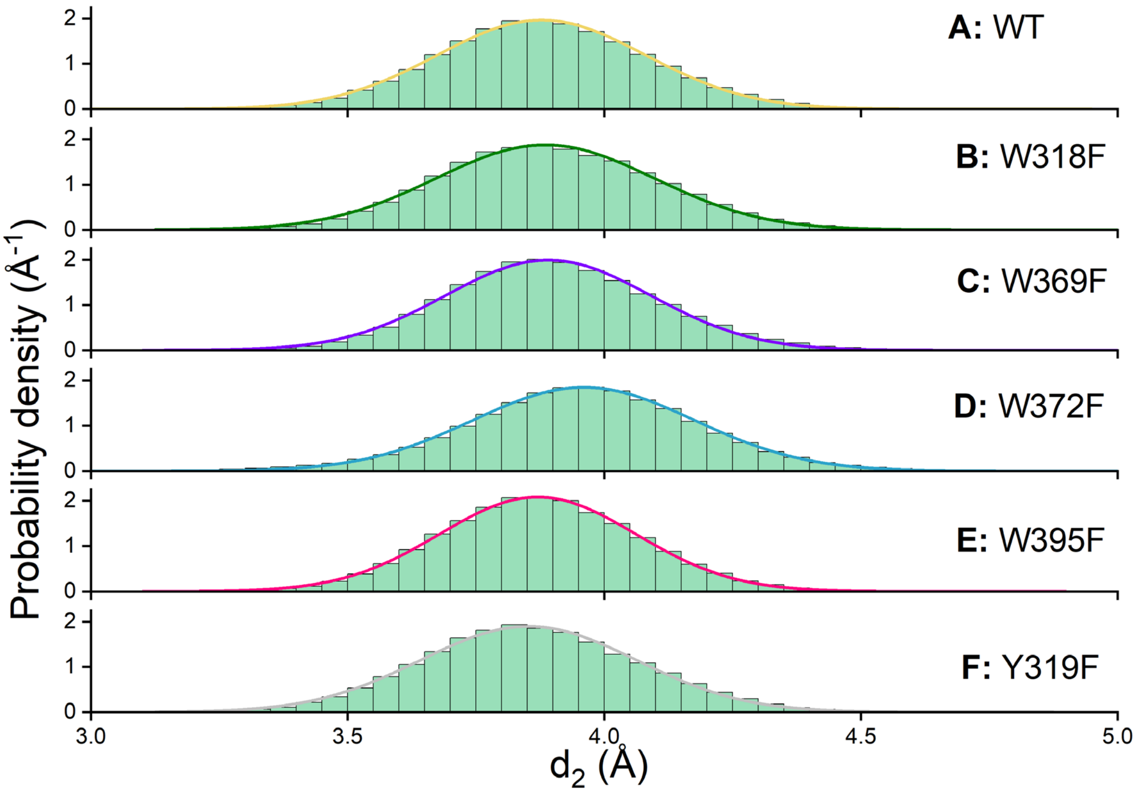

Figure S2: Probability distributions of the intramolecular contact (edge-to-edge) distance between W_A and W_B in ErCry4a, see Fig. 1. The distributions are shown for the wildtype ErCry4a (**A**) and mutations considered in this study (**B-F**). The distances were sampled every 5 ps of the production simulation yielding a total of 100,000 data points. The fitted distributions (lines) correspond to the single Gaussian fit functions, fitted utilising the Levenberg-Marquardt algorithm

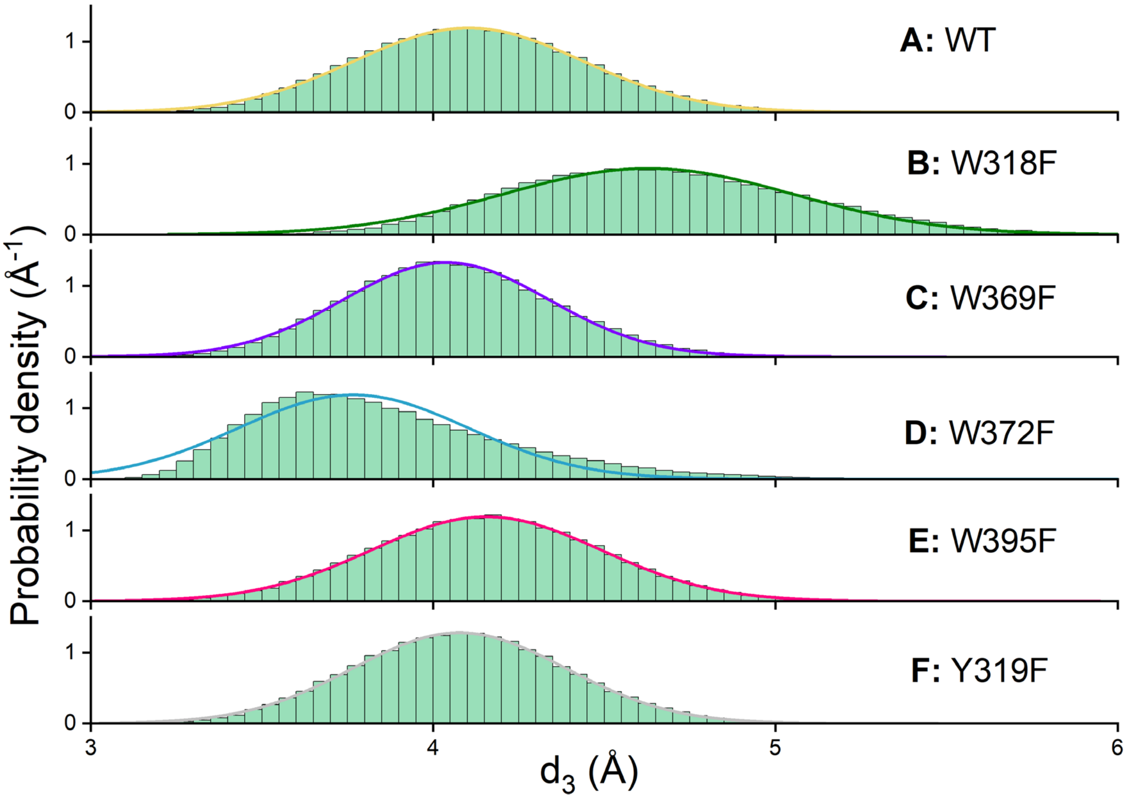

Figure S3: Probability distributions of the intramolecular contact (edge-to-edge) distance between W_B and W_C in ErCry4a, see Fig. 1. The distributions are shown for the wildtype ErCry4a (**A**) and mutations considered in this study (**B-F**). The distances were sampled every 5 ps of the production simulation yielding a total of 100,000 data points. The fitted distributions (lines) correspond to the single Gaussian fit functions, fitted utilising the Levenberg-Marquardt algorithm.

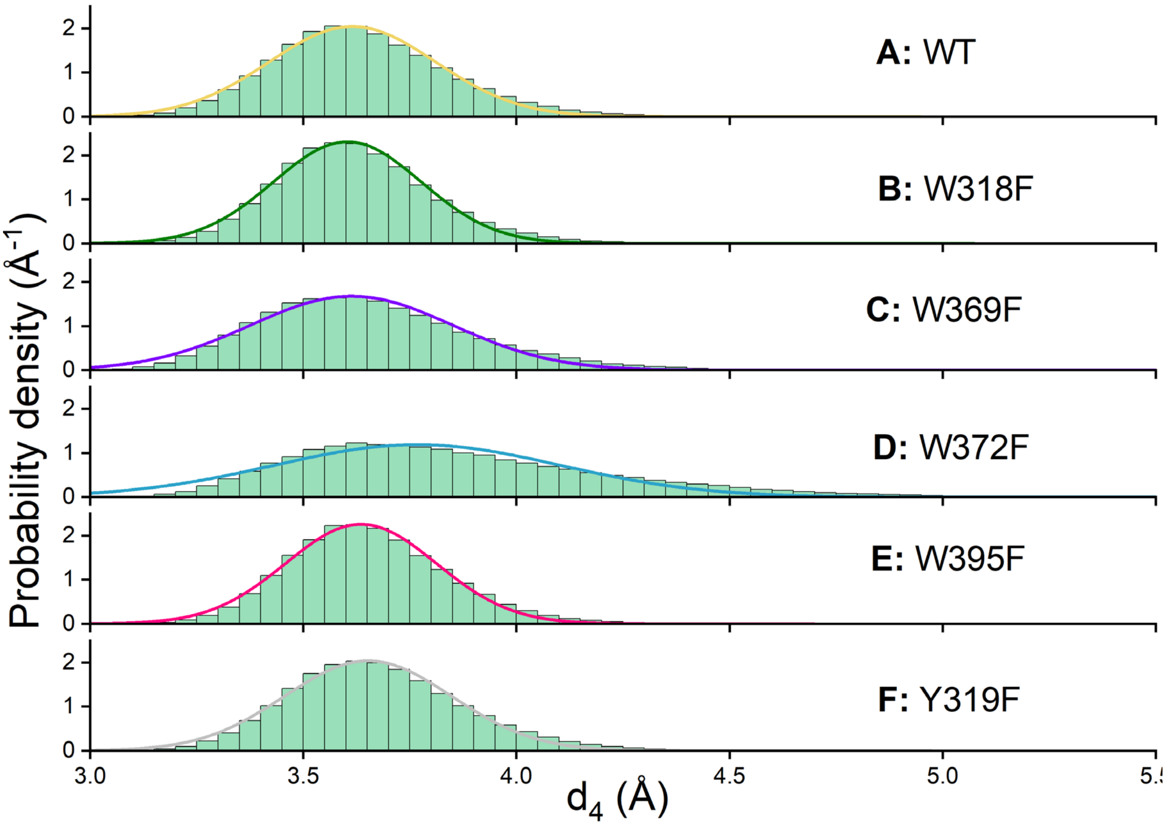

Figure S4: Probability distributions of the intramolecular contact (edge-to-edge) distance between W_C and W_D in ErCry4a, see Fig. 1. The distributions are shown for the wildtype ErCry4a (**A**) and mutations considered in this study (**B-F**). The distances were sampled every 5 ps of the production simulation yielding a total of 100,000 data points. The fitted distributions (lines) correspond to the single Gaussian fit functions, fitted utilising the Levenberg-Marquardt algorithm.

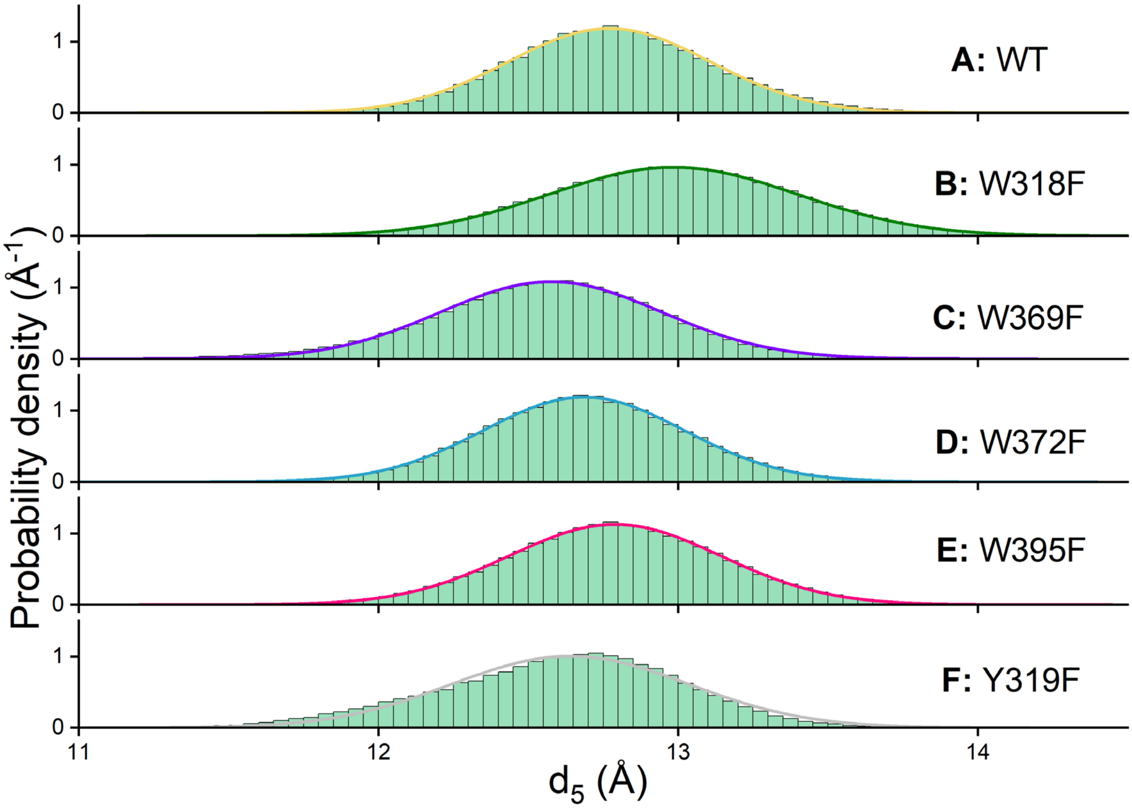

Figure S5: Probability distributions of the intramolecular contact (edge-to-edge) distance between FAD and W_C in ErCry4a, see Fig. 1. The distributions are shown for the wildtype ErCry4a (**A**) and mutations considered in this study (**B-F**). The distances were sampled every 5 ps of the production simulation yielding a total of 100,000 data points. The fitted distributions (lines) correspond to the single Gaussian fit functions, fitted utilising the Levenberg-Marquardt algorithm.

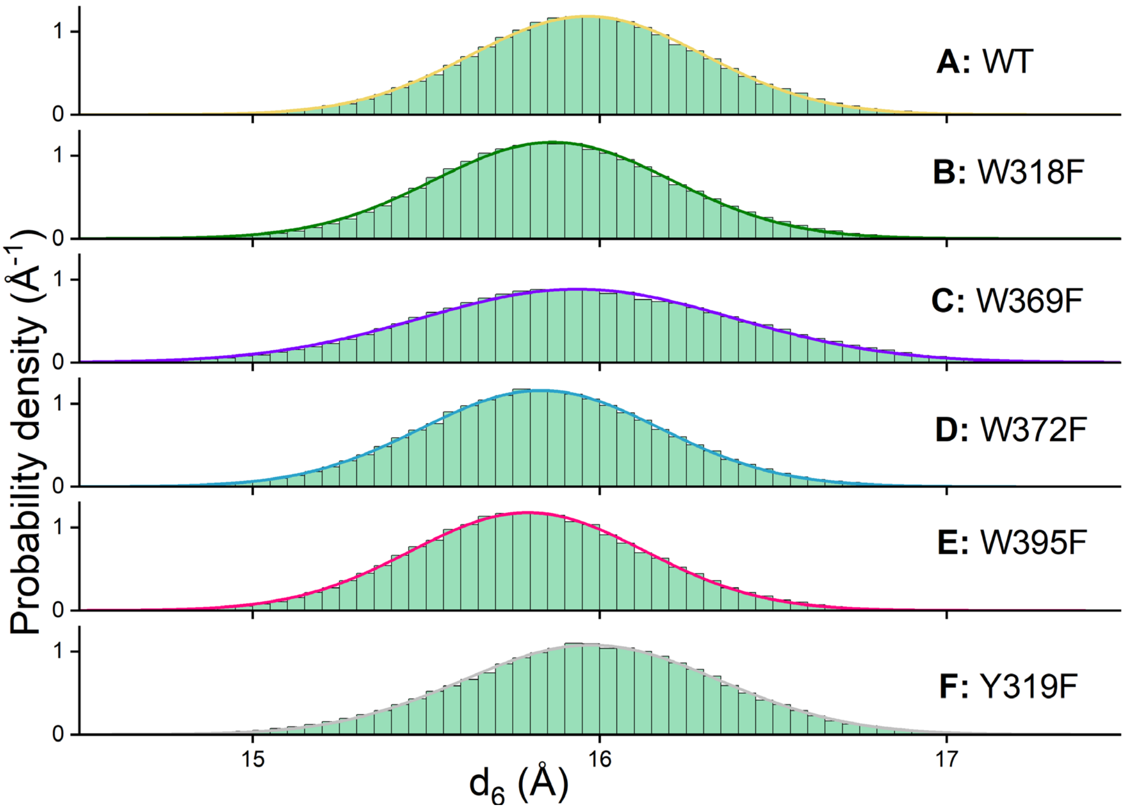

Figure S6: Probability distributions of the intramolecular contact (edge-to-edge) distance between FAD and W_D in ErCry4a, see Fig. 1. The distributions are shown for the wildtype ErCry4a (**A**) and mutations considered in this study (**B-F**). The distances were sampled every 5 ps of the production simulation yielding a total of 100,000 data points. The fitted distributions (lines) correspond to the single Gaussian fit functions, fitted utilising the Levenberg-Marquardt algorithm.

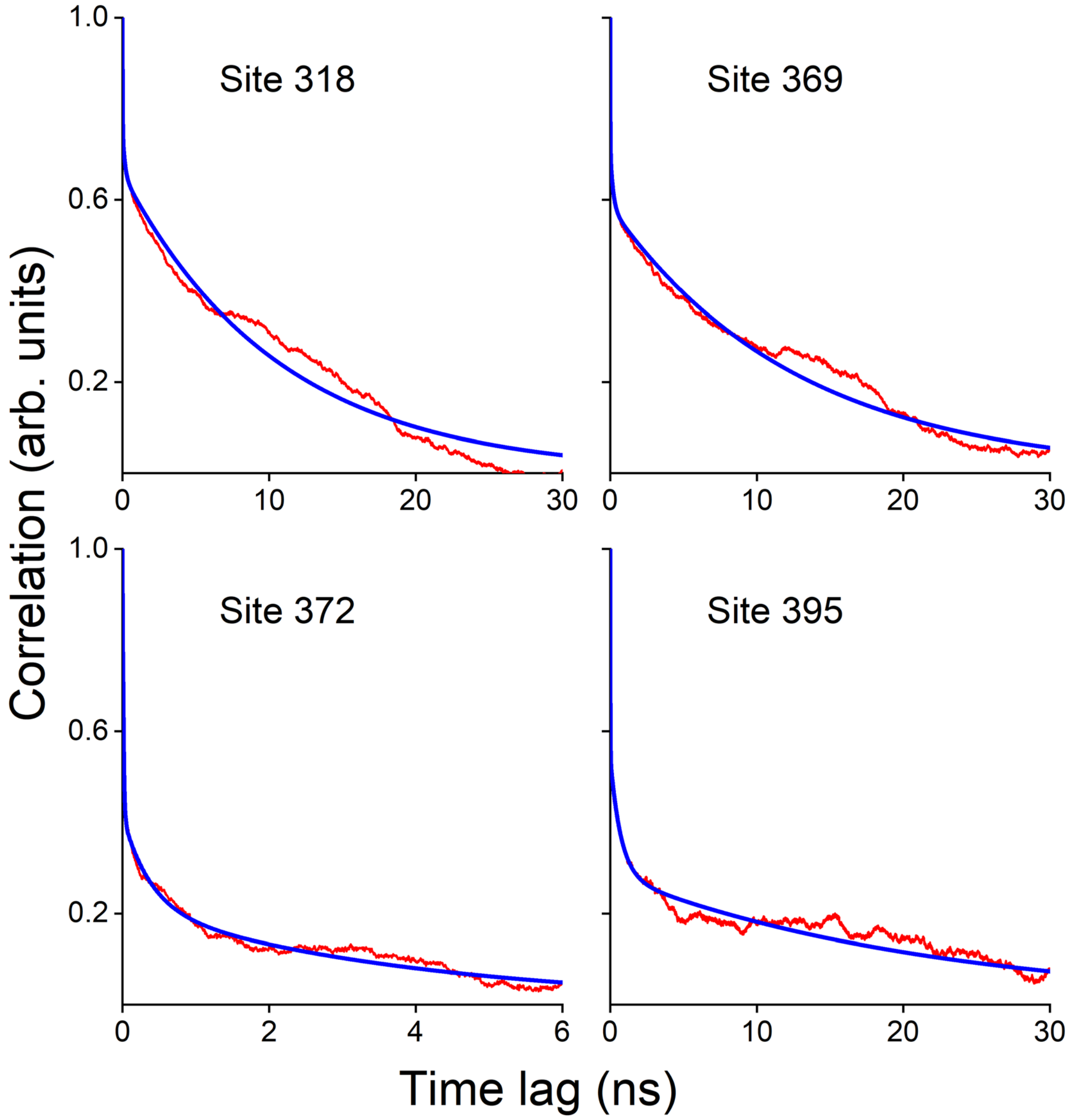

Figure S7: Time lag dependence of the correlation functions for the centre of mass motion of the four tryptophan sites inside ErCry4a WT and backbone atoms of surrounding amino acid residues within 10 Å (red line, Eq. (2)). The fitted dependencies (blue lines) correspond to the sums of three exponential decay functions, see, Eq. (3).

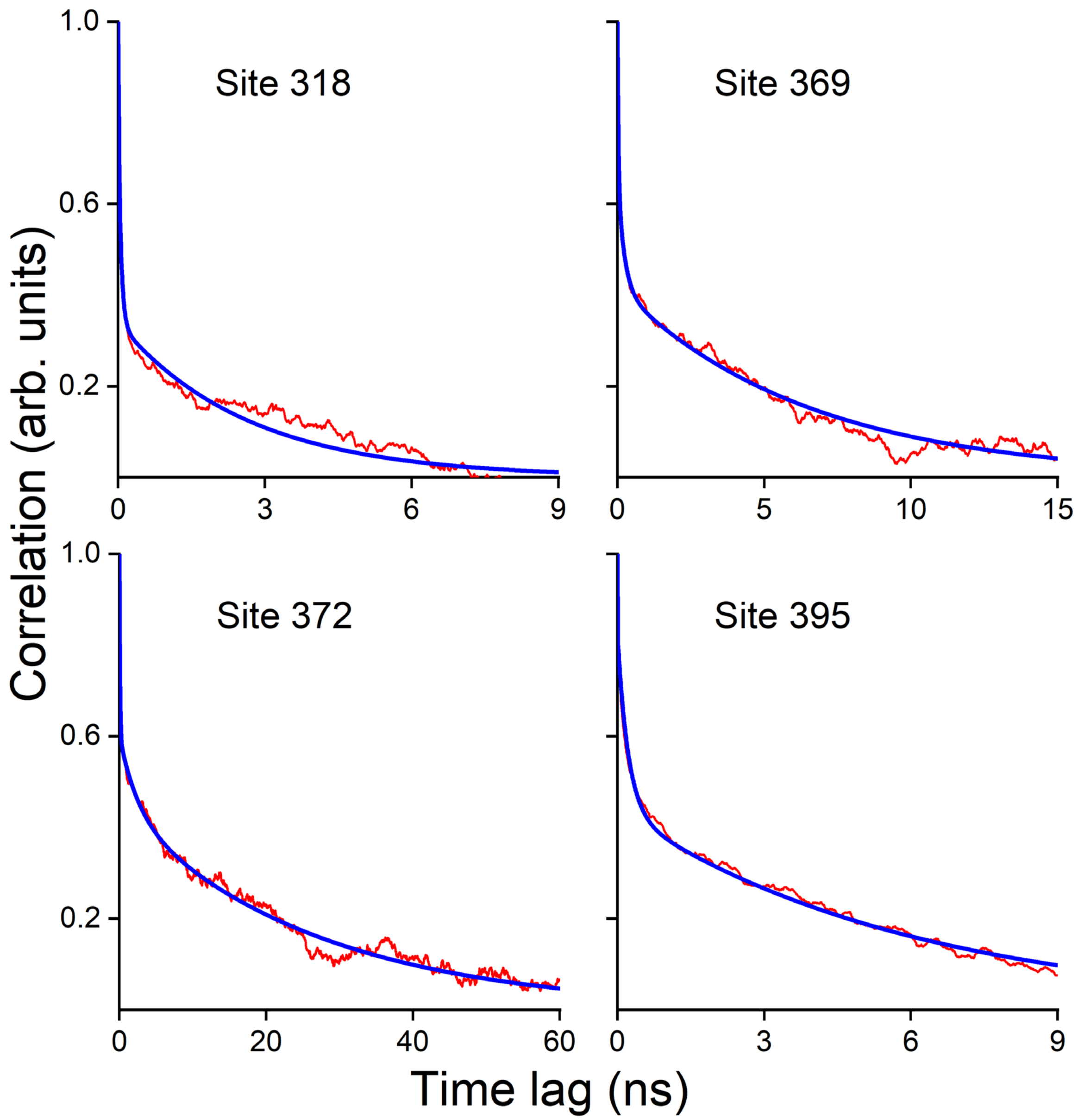

Figure S8: Time lag dependence of the correlation functions for the centre of mass motion of the four tryptophan sites inside ErCry4a W318F and backbone atoms of surrounding amino acid residues within 10 Å (red line, Eq. (2)). The fitted dependencies (blue lines) correspond to the sums of three exponential decay functions, see, Eq. (3).

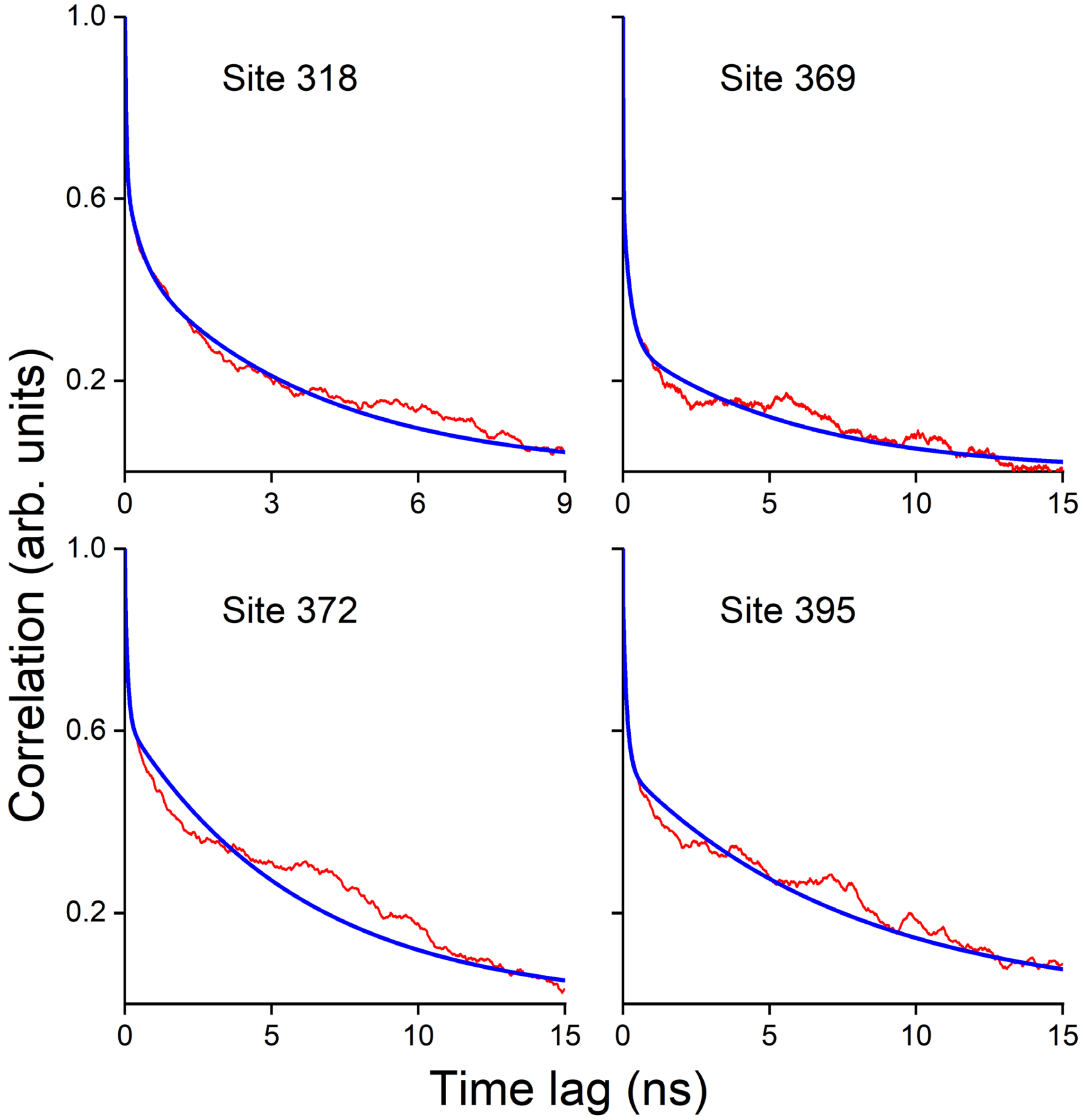

Figure S9: Time lag dependence of the correlation functions for the centre of mass motion of the four tryptophan sites inside ErCry4a W369F and backbone atoms of surrounding amino acid residues within 10 Å (red line, Eq. (2)). The fitted dependencies (blue lines) correspond to the sums of three exponential decay functions, see, Eq. (3).

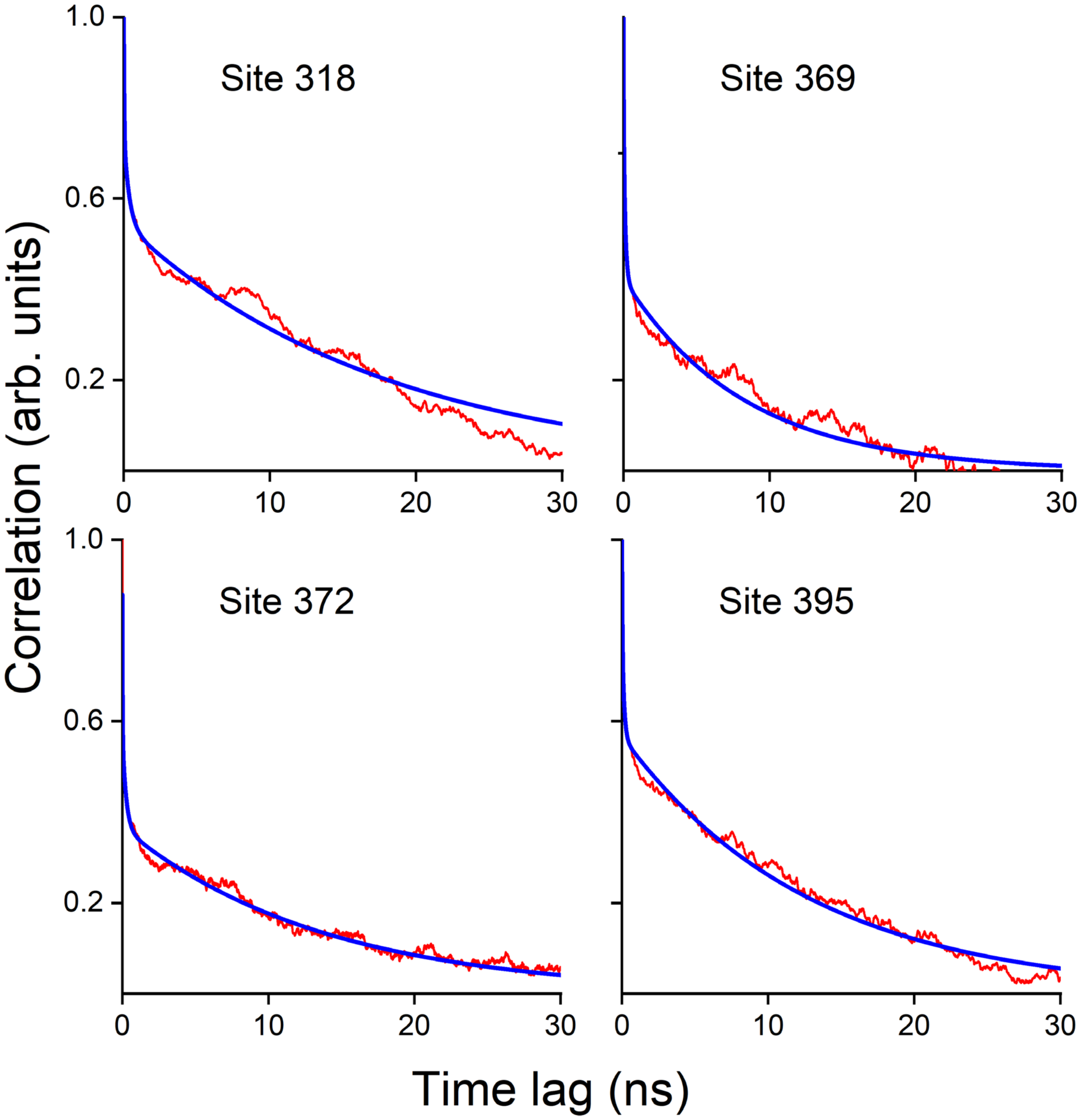

Figure S10: Time lag dependence of the correlation functions for the centre of mass motion of the four tryptophan sites inside ErCry4a W372F and backbone atoms of surrounding acid residues within 10 Å (red line, Eq. (2)). The fitted dependencies (blue lines) correspond to the sums of three exponential decay functions, see, Eq. (3).

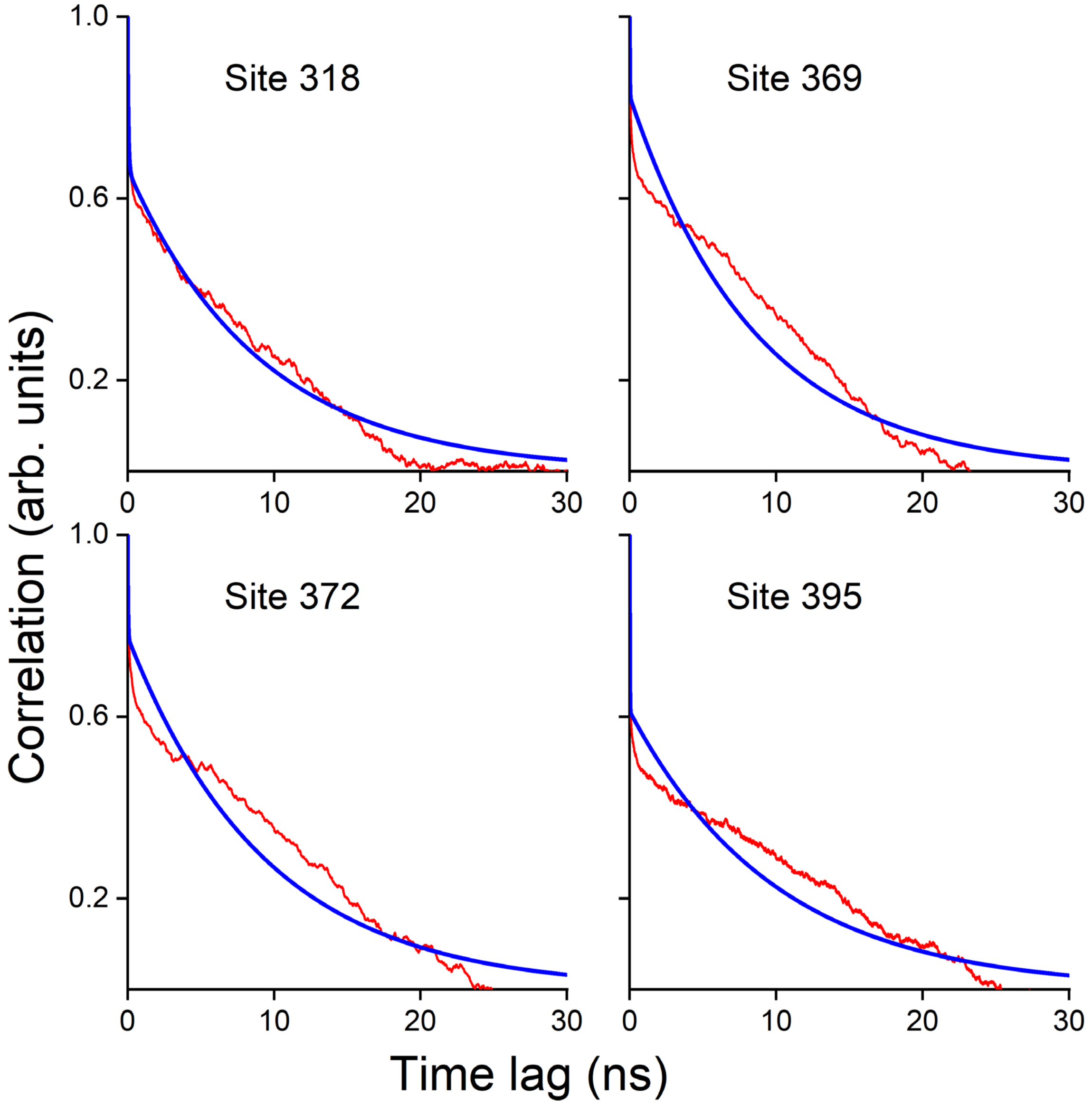

Figure S11: Time lag dependence of the correlation functions for the centre of mass motion of the four tryptophan sites inside ErCry4a W395F and backbone atoms of surrounding amino acid residues within 10 Å (red line, Eq. (2)). The fitted dependencies (blue lines) correspond to the sums of three exponential decay functions, see, Eq. (3).

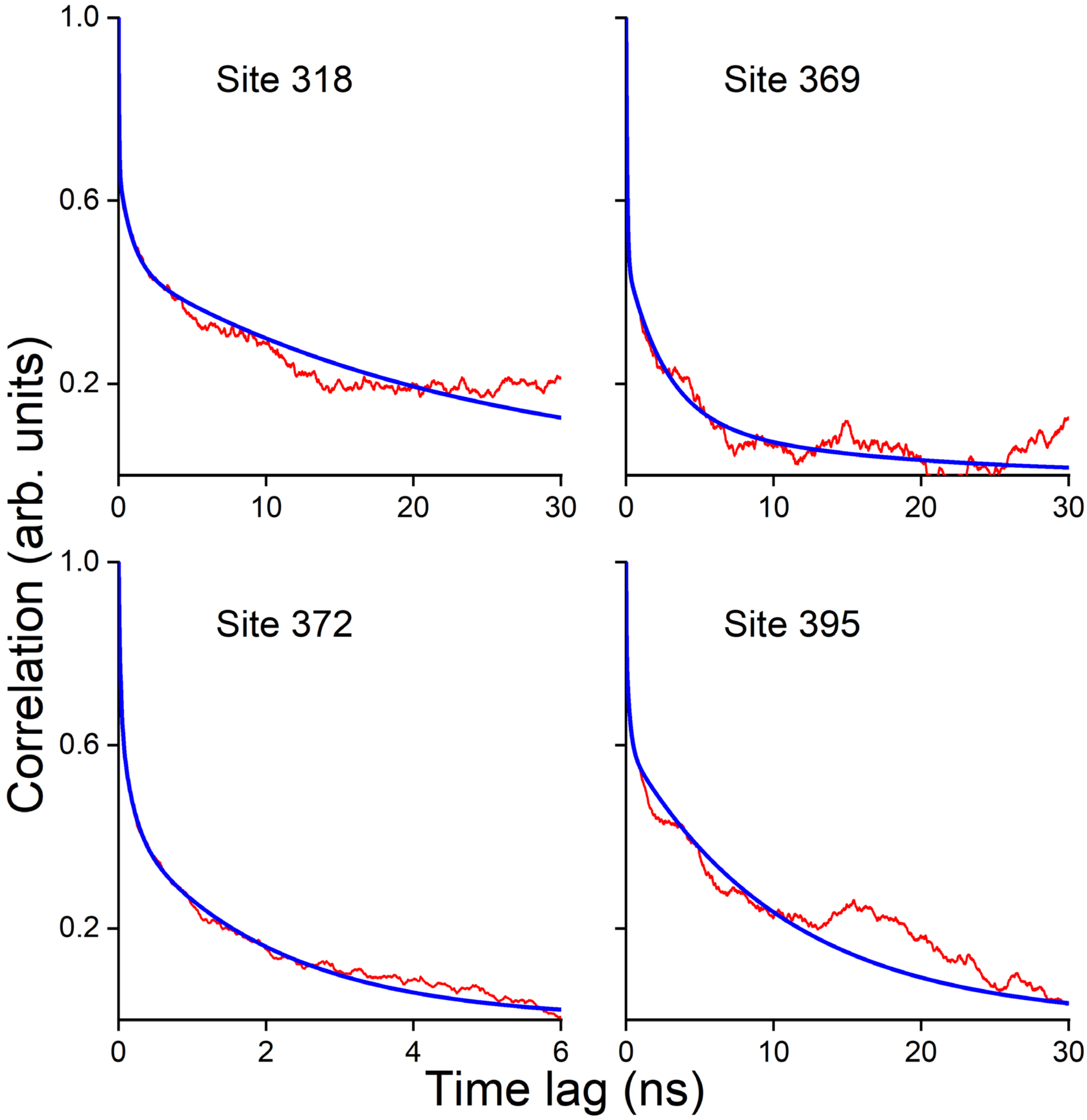

Figure S12: Time lag dependence of the correlation functions for the centre of mass motion of the four tryptophan sites inside ErCry4a Y319F and backbone atoms of surrounding amino acid residues within 10 Å (red line, Eq. (2)). The fitted dependencies (blue lines) correspond to the sums of three exponential decay functions, see, Eq. (3).

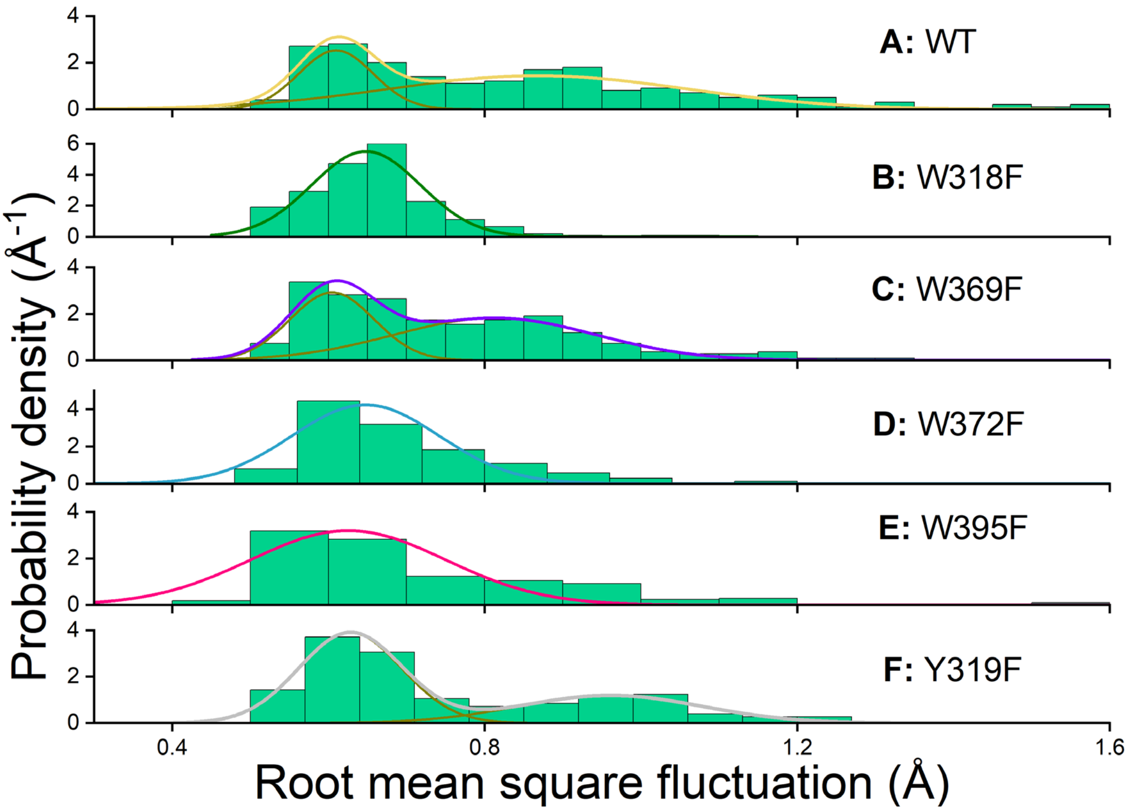

Figure S13: Probability distributions of the root mean square fluctuations arising in ErCry4a WT and its mutants (see labels in the plots) at the site 318. The fluctuations of the backbone atoms of residues 313-323 were analysed. The histograms show the bootstrapped data available from the simulations (see main text), while the lines correspond to the Gaussian fits. The residual sum of squares, r^2, for the fits is provided in Table 2, while the standard deviation of the fits is used to define the error bars in Fig. 5. In some cases, a double Gaussian fit was employed to better match the data.

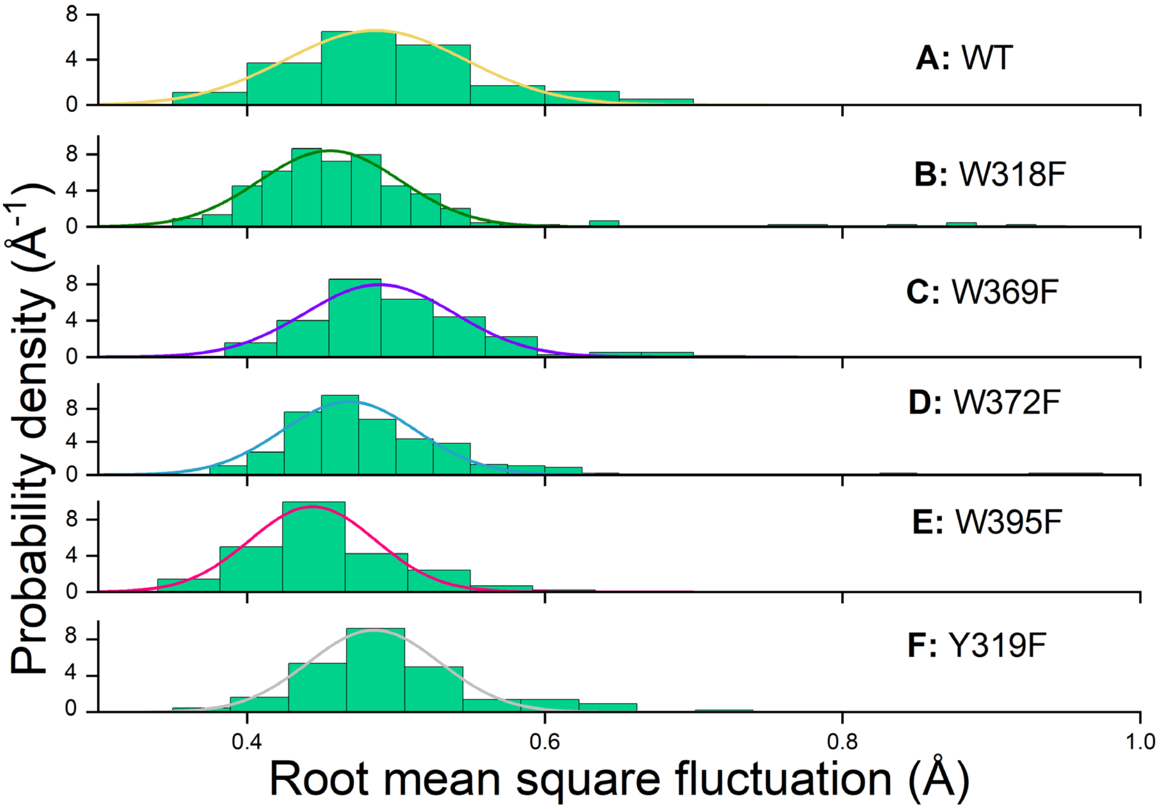

Figure S14: Probability distributions of the root mean square fluctuations arising in ErCry4a WT and its mutants (see labels in the plots) at the site 369. The fluctuations of the backbone atoms of residues 364-374 were analysed. The histograms show the bootstrapped data available from the simulations (see main text), while the lines correspond to the Gaussian fits. The residual sum of squares, r^2, for the fits is provided in Table 2, while the standard deviation of the fits is used to define the error bars in Fig. 5.

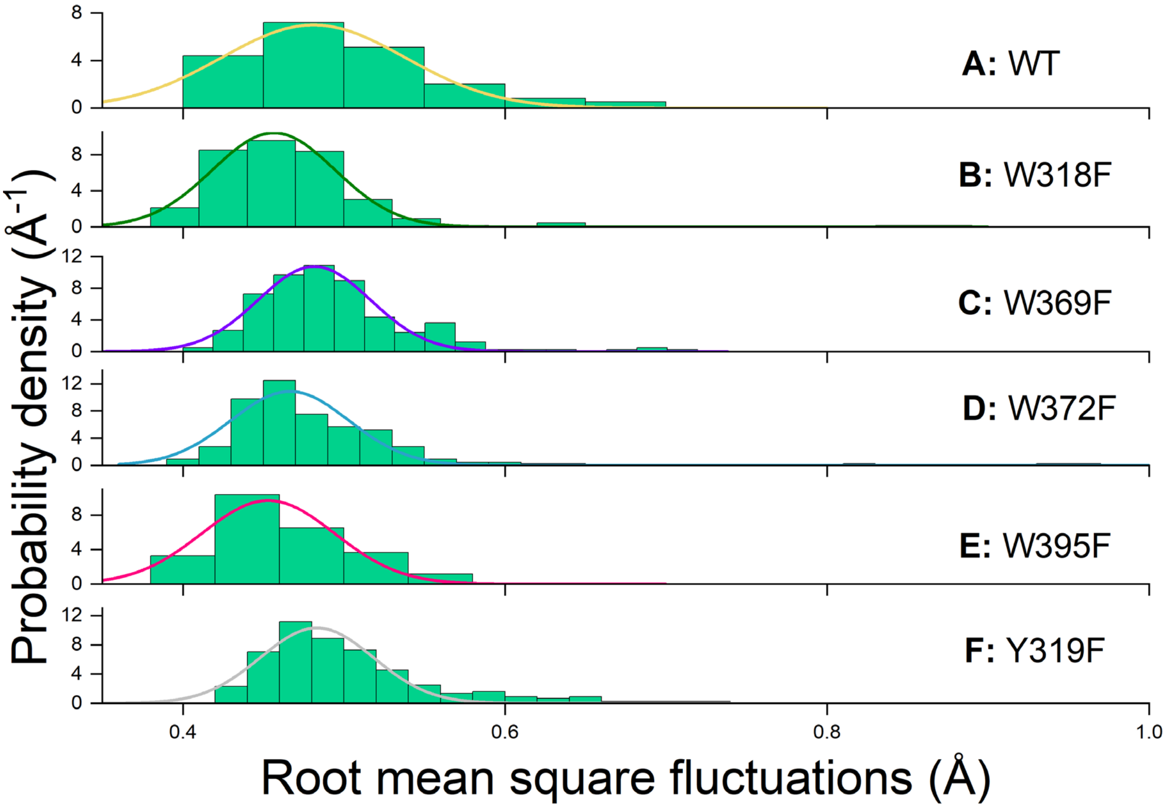

Figure S15: Probability distributions of the root mean square fluctuations arising in ErCry4a WT and its mutants (see labels in the plots) at the site 372. The fluctuations of the backbone atoms of residues 367-377 were analysed. The histograms show the bootstrapped data available from the simulations (see main text), while the lines correspond to the Gaussian fits. The residual sum of squares, r^2, for the fits is provided in Table 2, while the standard deviation of the fits is used to define the error bars in Fig. 5.

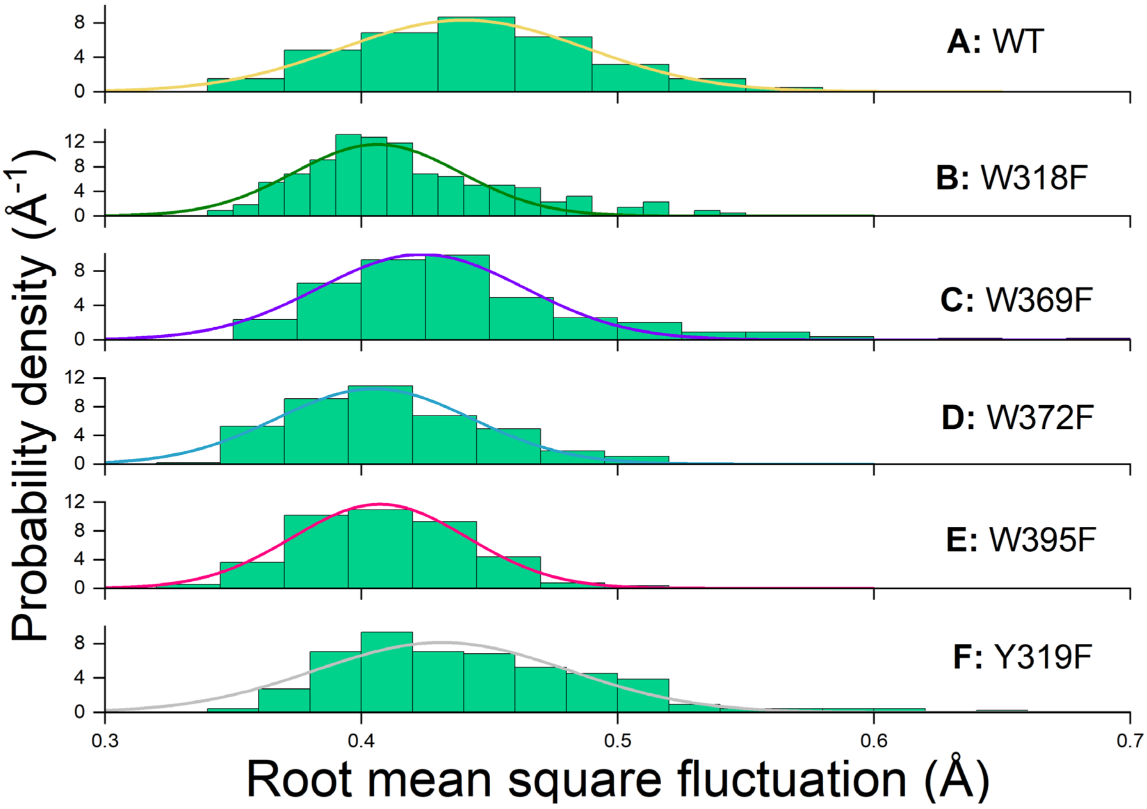

Figure S16: Probability distributions of the root mean square fluctuations arising in ErCry4a WT and its mutants (see labels in the plots) at the site 395. The fluctuations of the backbone atoms of residues 390-400 were analysed. The histograms show the bootstrapped data available from the simulations (see main text), while the lines correspond to the Gaussian fits. The residual sum of squares, r^2, for the fits is provided in Table 2, while the standard deviation of the fits is used to define the error bars in Fig. 5.
